# An efficient brain tumor classification system with accurate recognition and complementary downsampling blocks

**DOI:** 10.1371/journal.pone.0343457

**Published:** 2026-09-30

**Authors:** Jincan Zhang, Chuanqi Cai, XingHua Tan, Wenna Chen, Xin Zhao, Ganqin Du, Hongwei Jiang

**Affiliations:** 1 College of Information Engineering, Henan University of Science and Technology, Luoyang, China; 2 The First Affiliated Hospital, and College of Clinical Medicine of Henan University of Science and Technology, Luoyang, China; University of Manitoba, CANADA

## Abstract

Due to the substantial impact of brain tumors on human health, precise identification of tumor types is vital for patient prognosis and guiding treatment decisions. Magnetic Resonance Imaging (MRI) technology, owing to its non-invasive nature and high resolution, is indispensable in accurately classifying brain tumor types. However, manual interpretation of MRI images is not only time-consuming and labor-intensive but also susceptible to subjective bias. Hence, many automatic brain tumor diagnosis systems utilizing deep learning techniques have been developed. This paper introduces an advanced modularized Convolutional Neural Network (CNN) that incorporates ConvDAtt block, Complementary Information (CI) downsampling block, and Spatial Pyramid Pooling (SPP) block. Firstly, the ConvDAtt block combines convolution, depth-separable convolution and Coordinated Attention (CA) mechanism. The module alleviates the gradient vanishing problem in deep neural network training, enhancing the training stability and performance of the model. In the ConvDAtt block, the CA mechanism can not only capture information across channels, but also sense direction and position sensitive information, improving the accuracy of the model’s identification of objects of interest. Secondly, the CI block is a downsampling block with convolution and pooling functions. In the CI block, there is a complementary relationship between the pooling layer and the convolution layer, which is mainly reflected in two aspects, feature extraction and dimensionality reduction, enlargement of receptive field. Moreover, the complementary relationship can solve the problem of information loss in the process of downsampling, while also significantly reducing computational cost. Thirdly, the SPP block extracts features from different scales of the input data and integrates these features, thus enhancing the performance of the network, and enhancing the robustness of the model. The combination of ConvDAtt block, CI block and SPP block can effectively extract the information features and discriminant features of brain tumor MRI images, which has the advantages of parameter reduction, stable training and clear structure.

## 1. Introduction

Brain tumors are primarily categorized into two distinct types, primary brain tumors and brain metastases. Surgical intervention, particularly complete resection, is often regarded as the optimal approach for managing this condition [1]. Prior to treatment, a thorough evaluation of the tumor is imperative to ensure the effectiveness of the therapeutic intervention. MRI, a widely utilized imaging technique for elucidating the shape, size, and location of brain tumors, holds a crucial role in this assessment [2]. However, the analysis of MRI images requires professional expertise, rendering it a potentially error-prone process. Therefore, research on automatic diagnosis of brain tumors is of great significance to patients, doctors, technicians and hospitals [3].

Traditional Machine Learning (ML) algorithms have long been prevalent in the medical field and widely employed [4]. For example, Twinkle Bansal et al. [5] have combined the Grayscale Covariance Matrix (GLAM) technique with shape features to comprehensively and accurately extract mixed tumor region characteristics and a hybrid classification strategy incorporating Random Forest (RF), K-Nearest Neighbors (KNN), and Decision Tree (DT) classifiers has been employed to enhance classification accuracy and stability. In [6], the Gray Level Co-occurrence Matrix (GLCM) and Gray Level Run Length Matrix (GLRLM) were utilized to extract image features. Then, a Probabilistic Neural Network (PNN) classifier was utilized to categorize MRI brain images. In [7], multiple supervised machine learning were compared in depth to assess their actual effectiveness for internal effectiveness and accuracy in predicting clinical events. In [8], ML algorithms have been applied to predict the development of cancer symptoms and have successfully identified predictors of these symptoms. In [9], the categorization of brain tumors was successfully accomplished through the integration of various techniques, Discrete Wavelet Transform (DWT), Principal Component Analysis (PCA), Feedforward Backpropagation Artificial Neural Network (FP-ANN), and the KNN classifier. These algorithms heavily rely on hand-crafted features, resulting in time-consuming processes prone to errors [10].

Convolutional Neural Networks(CNNs) have effectively addressed this issue by autonomously learning invariant features, showcasing superior performance and gaining wide application [11]. Recently, CNNs have yielded outstanding outcomes in brain tumor classification. In [12], a CNN is used to classify primary brain tumors. To screen out the best-performing models, a series of experiments were designed and conducted. Finally, a CNN architecture with an overall performance of 96% was successfully built. Roy et al. [13] conducted in-depth comparative analysis of simple artificial neural networks including basic CNNs and extended CNNs. The experimental results show that extended CNN exhibits excellent performance in classification tasks, with an accuracy rate of 97%. G. Vidya Sagar et al. [14] introduced a residual learning technique tailored for brain tumor classification, involving three core steps, preprocessing, feature extraction, and classification. In [15], a technique for training CNN was introduced, employing a class-weighted focal loss approach. In addition, in order to further improve the effectiveness of the classifier, this study utilized the integrated features of multiple CNN models. In [16], the proposed framework adopted transfer learning, wherein deep features were extracted from MRI images using a pre-trained deep convolutional neural network. Subsequently, these features undergone evaluation with multiple machine learning classifiers, and the top three best-performing classifiers were selected and integrated into a deep feature set to enhance feature representation and classification accuracy. In [17], a stacked modeling strategy was proposed, which significantly improved the final accuracy of the model and effectively overcame the challenge of insufficient labeled training data. Three CNN architecture models namely VGG19, Inception V3, and ResNet101 have been adequately trained on medical image datasets and were used in this study. Khan et al. [18] proposed a model that adds three additional layers to the MobileNet backbone and integrates residual learning, while replacing the ReLU activation function with Swish to improve information retention and feature representation. This model achieved an accuracy of 96.94% on the Figshare multi-class classification dataset.

In order to further improve the accuracy of the model, many novel models have appeared in recent years. For example, in [19], DenseNet201, ResNet152V2, and InceptionResNetV2 were utilized as pre-trained models, forming a stacked ensemble model to enhance generalization ability. In [20], six optimization algorithms were combined with two classifiers, softmax and Support Vector Machine (SVM). The best performance was observed when the softmax classifier was combined with the Nesterov Accelerated Adaptive Moment Estimation (Nadam) optimizer. Muhammad Irfan et al. [21] used multiple pre-trained models (ResNet50, VGG16, VGG19 and U-Net) to classify brain tumors or no tumors. Among the pre-trained models compared, the U-Net model showed excellent performance with an accuracy of 95%. Bala Venkateswarlu Isunuri et al. [22] proposed an Ensemble Coupled Convolutional Network (ECCN) model designed specifically for classifying three distinct types of brain tumors. This proposed model was evaluated on the REMBRANDT dataset, demonstrating an accuracy rate of 96.95%. Gomez-Guzman et al. [23] conducted an evaluation of seven deep convolutional neural network (CNN) models in the context of brain tumor classification, with one model being generic and the remaining six being pre-trained. Amongst all the models compared, InceptionV3 emerged as the superior model, achieving an impressive average accuracy of 97.12%. Trisheela et al. [24] utilized VGGNet for feature extraction, employed Invasive Weed Optimization (IWO) to select the most relevant features, and utilized an enhanced DarkNet-53 deep learning (DL) model to classify brain tumors. The model achieved an accuracy of 95% on a dataset consisting of 3,264 MRI scan images. Tariq et al. [25] achieved an accuracy rate of 96% on the brain tumor MRI dataset by combining EfficientNetV2 for efficient feature extraction and Vision Transformer (ViT) for capturing global contextual information, and applying geometric mean ensemble learning to integrate the predictions from both models.

Clinically, the precise identification of brain tumor types directly informs treatment planning, surgical strategy, and prognostic evaluation. However, manual MRI interpretation is labor-intensive and subject to inter-observer variability, which poses significant challenges in time-sensitive clinical environments. Therefore, developing an automated and reliable classification system holds great potential to address these challenges in clinical practice. In this paper, we propose a modular deep convolutional neural network specifically designed for high-accuracy, multi-class brain tumor classification. The network incorporates three key components: the ConvDAtt block, which combines convolution, depth-separable convolution, coordinate attention (CA), and residual connections to enhance feature extraction and training stability [26], the Complementary Information (CI) downsampling block, which exploits the complementary strengths of convolution and pooling to preserve rich feature information during downsampling, and the Spatial Pyramid Pooling (SPP) block, which captures multi-scale spatial information and aggregates it into a fixed-length feature vector [27]. This modular design not only achieves state-of-the-art classification accuracy but also significantly reduces computational overhead, thereby serving as a promising methodological framework that could potentially inform future clinical decision-support systems, pending rigorous prospective validation in real-world settings. The effectiveness of the proposed method is extensively validated on two public benchmark datasets, consistently demonstrating substantial advantages over existing approaches. It introduces an efficient modular brain tumor classification network [28] that improves classification accuracy through the synergistic interaction of multiple modules, showing strong potential as a reliable auxiliary system for computer-aided diagnosis. The contribution of this research includes the following aspects:

- A novel modular convolutional neural network is proposed for brain tumor image classification from MRI.
- The ConvDAtt block, integrating convolution, depth-separable convolution, CA mechanism, and residual connectivity, is proposed to enhance network performance and training efficiency. The introduction of CA mechanism [29] helps the network to extract key features from the original data, thus improving the performance of the model.
- The CI downsampling block leverages the complementary strengths of convolution and pooling to preserve rich spatial information during downsampling, while also substantially improving computational efficiency.
- The SPP block is employed to capture multi-scale spatial information from input feature maps, which further strengthens the model’s robustness against image quality degradation.
- The proposed method demonstrates superior effectiveness and accuracy compared to prior art methods, as evidenced by experimental results using two public datasets.

The structure of this article is as follows: Section 2 elaborates on the proposed method in detail, Section 3 presents the experimental results, Section 4 conducts an in-depth discussion of the results, and Section 5 summarizes the article.

## 2. Materials and methods

### 2.1. Datasets

This paper conducts a series of experiments aimed at classifying brain tumors using publicly available MRI datasets. The dataset, sourced from Cheng et al.[30], comprises 3064 grayscale brain MR images categorized into three groups, 708 meningioma samples, 1426 glioma samples, and 930 pituitary tumor samples. In addition, experimental data sets consisting of 3264 grayscale brain MR images from the Kaggle platform [31] were also used. This dataset categorizes images into four groups, meningiomas, gliomas, pituitary tumors and normal tumor-free samples, with respective sample counts of 940, 926, 901 and 497. The inclusive categorization encompasses normal tumor-free samples, augmenting the study’s utility. Representative images are depicted in Fig 1, showcasing various brain tumor morphologies and characteristics. For model assessment, each dataset is split into training and testing sets in an 80/20 ratio to ensure sufficient data for model learning and independent validation. Table 1, showing details of the two datasets, provides a clear basis for subsequent analysis and discussion.

**Table 1:** Details of datasets.

Dataset	Classes	Number of each class	Total
Chen	Glioma	1426	3064
	Meningioma	708	
	Pituitary	930	
Kaggle	Glioma	926	3264
	Meningioma	940	
	Pituitary	901	
	No tumor	497	

**Fig 1:**
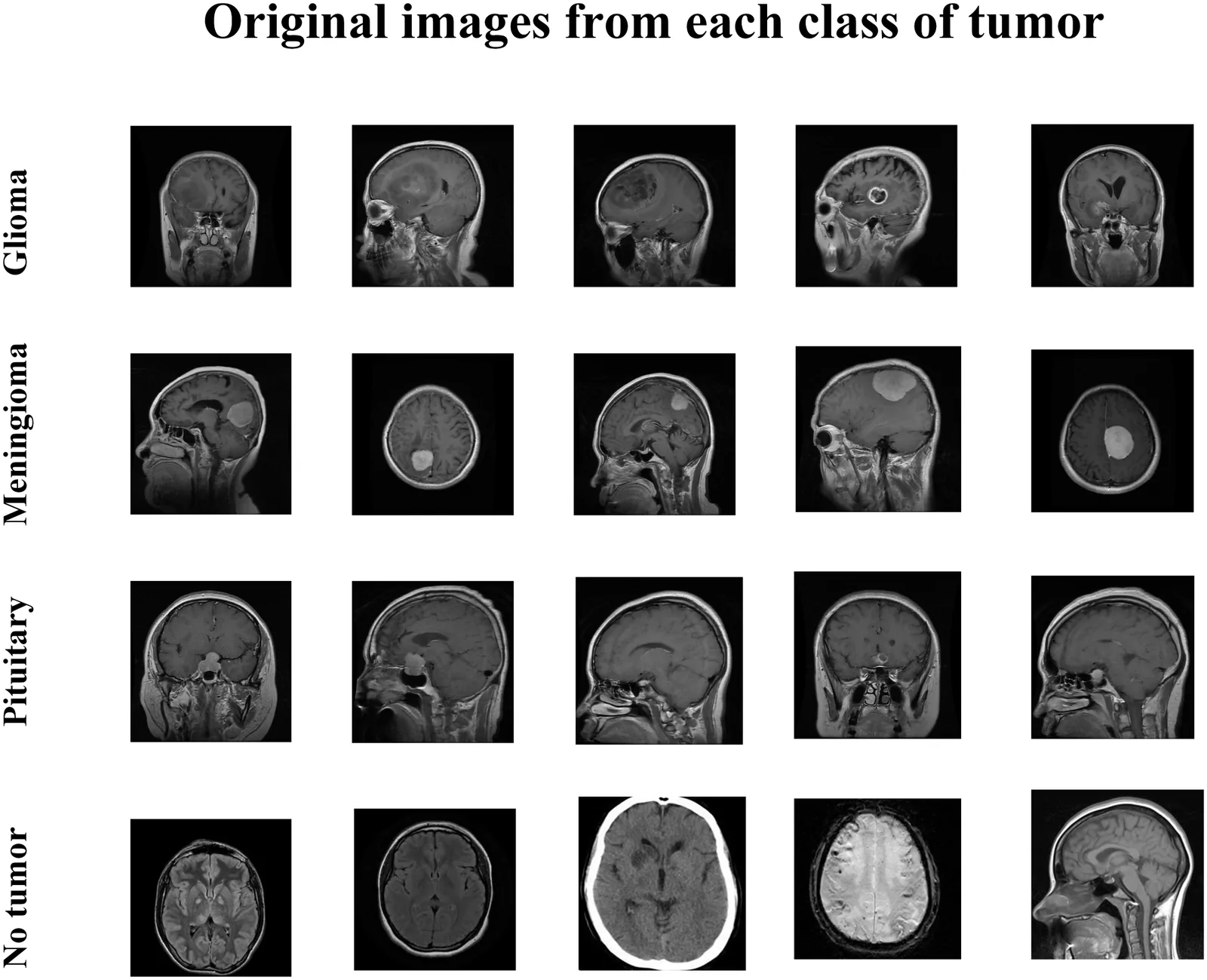
Raw magnetic resonance images of the Kaggle dataset.

### 2.2. Preprocessing

The Chen dataset comprises grayscale images uniformly sized at 512 × 512 pixels. To ensure compatibility and computational efficiency, preprocessing was conducted [32]. Initially, all images were resized uniformly to 224 × 224 pixels, aligning them with the input requirements of the deep learning model. Subsequently, considering the model’s expectation of receiving three-channel RGB input, the original grayscale images were transformed into three-channel RGB format by duplicating the grayscale values across the RGB channels. For the Kaggle dataset, inconsistencies exist in image size and color space, with some being grayscale and others RGB. To ensure data consistency and model training stability, similar preprocessing steps were applied. Specifically, all images were uniformly resized to 224 × 224 pixels, and grayscale images were converted to the three-channel RGB format. This standardization of image size and color space across both datasets facilitates subsequent deep learning model training.

### 2.3. Classification system

The proposed brain tumor classification network, designed with a modular approach, efficiently captures informative and discriminative features from brain tumor MRI images. The network is specifically constructed to preserve significant spatial features in brain MRI images, ensuring the integrity and accuracy of essential information. Considering the importance of key features and capturing multi-scale spatial information in brain tumor MRI image classification, the CA mechanism and spatial pyramid pool are utilized to further improve classification accuracy. The integration enables the network to better discern and differentiate between different types of brain tumors.

Fig 2 illustrates the overarching framework of the proposed brain tumor classification system. The dataset is divided explicitly into training and testing sets, with no overlap between them. The model is trained on the training set, and the trained model is saved to the disk. Subsequently, it loads the saved model to classify the testing set and evaluates its classification performance.

**Fig 2:**
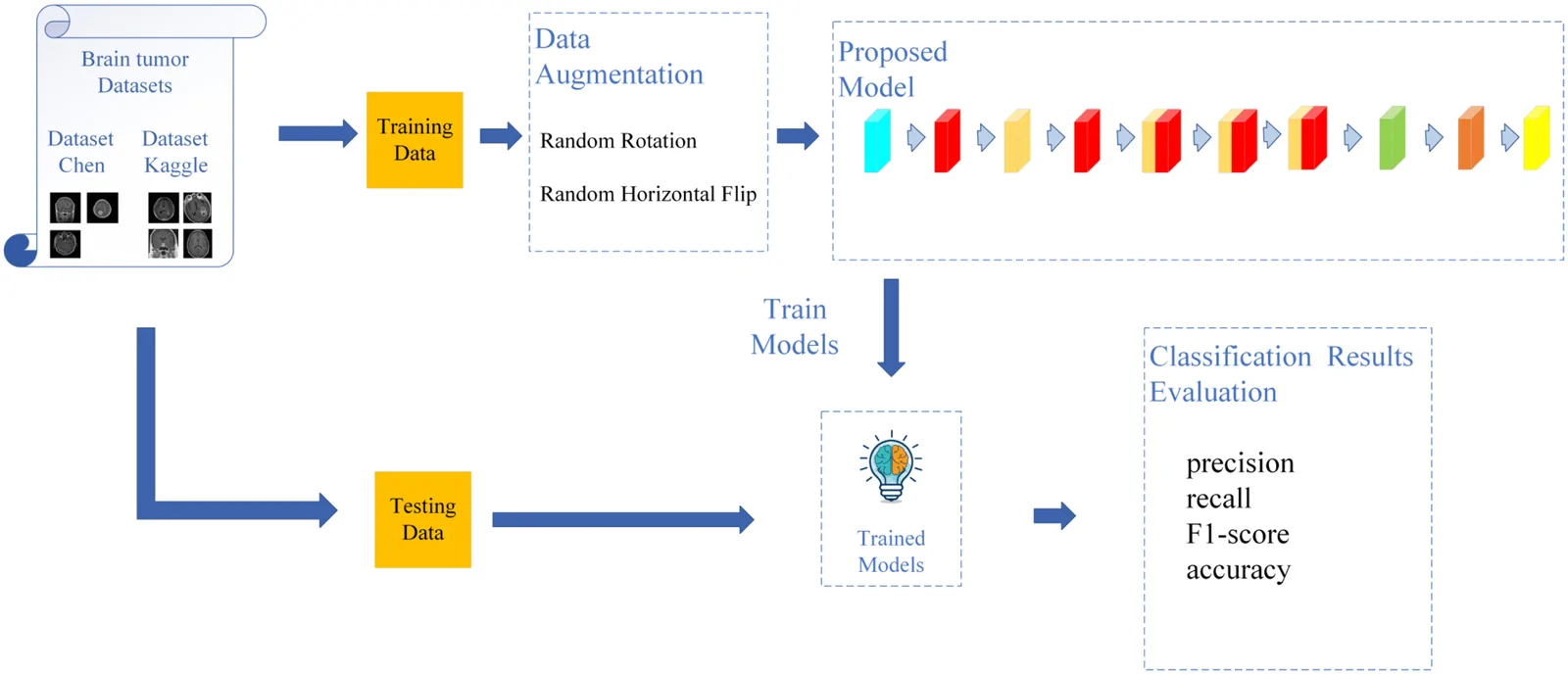
Framework of the proposed brain tumor classification system.

Fig 3 depicts the schematic diagram of the proposed network architecture. Initially, the number of feature channels is increased by sequential convolutional layers (referred to as conv blocks). Subsequently, the spatial dimensions of the feature maps are reduced by the CI layer. Following this, the ConvDAtt layer conducts feature extraction and transformation, followed by further reduction in feature map size with the CI layer. Subsequently, the network undergoes three iterative cycles, facilitating additional feature fusion and downsampling. The SPP layer then captures multi-scale spatial information from the input feature mapping. This layer maps the feature pool into a fixed-size output. Finally, the flattened feature maps are fed into a fully connected layer for classification. The ConvDAtt layer can effectively extract features and enhance the network’s focus on important features. Secondly, the CI layer realizes the size reduction and feature fusion of the feature graph, which not only preserves the spatial details, but also enriches the feature representation, which is crucial to improve the performance of the neural network. In sections 2.31 and 2.32, the functionality and operations of two key layers, the ConvDAtt layer and the CI layer, are explored.

**Fig 3:**
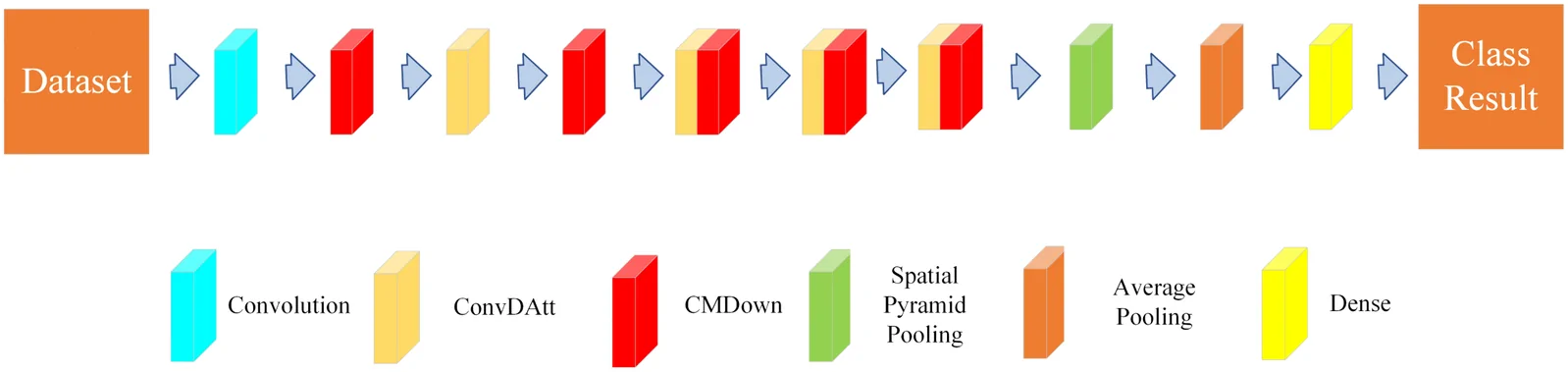
The proposed model.

#### 2.3.1. ConvDAtt block.

The ConvDAtt module is illustrated in Fig 4. The input data first passes through Conv1, followed by depth-separable convolution, the CA attention mechanism, Conv2, and finally a residual connection.

**Fig 4:**
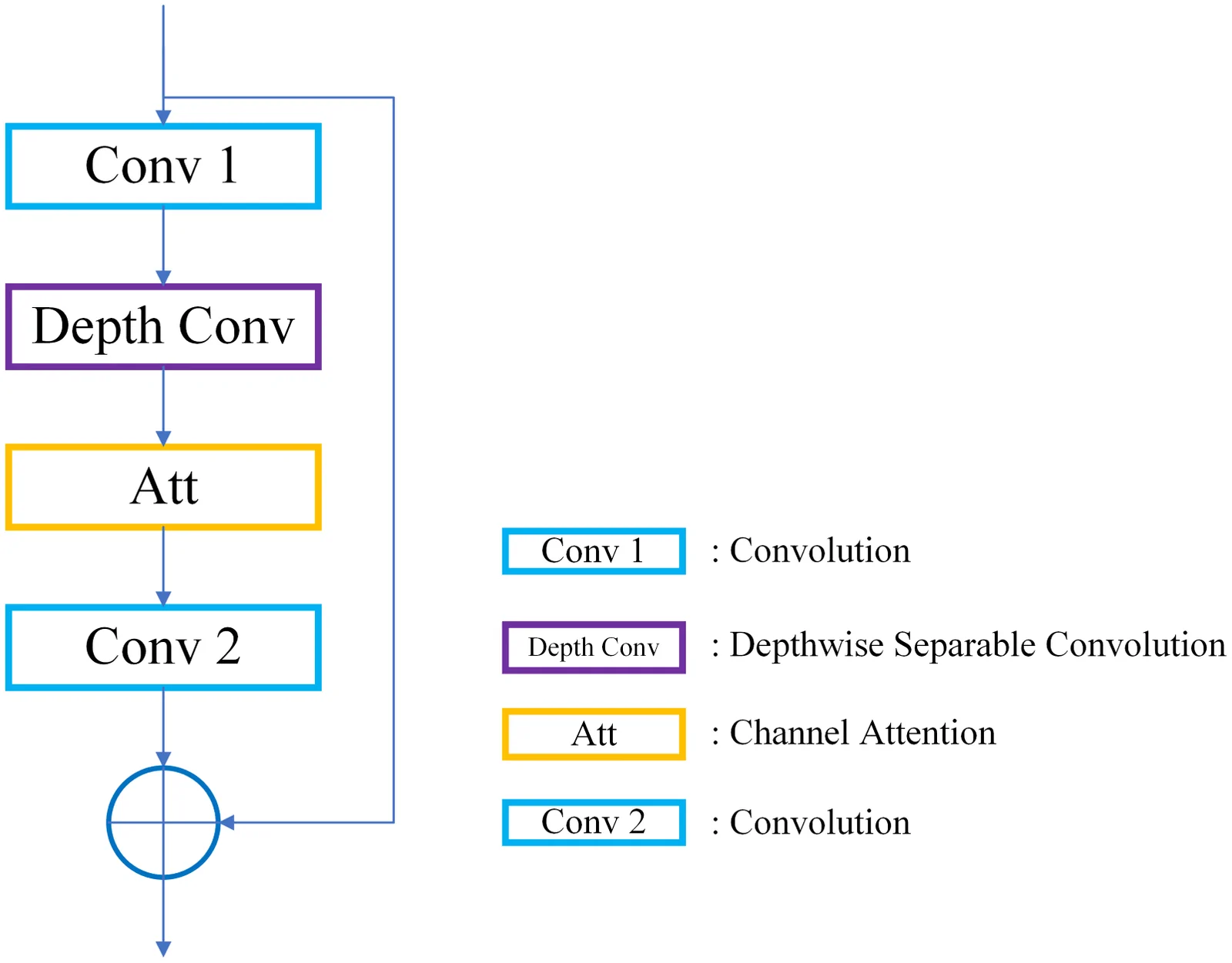
ConvDAtt block.

Initially the Conv1 layer is used. This convolutional layer is designed to extract effective features from the input data. Subsequently, a depth-separable convolution is used to reduce the number of parameters and improve the computational efficiency of the model.

Subsequently, the Coordinate Attention (CA) mechanism is employed. Because traditional attention mechanisms (e.g., SENet, ECANet) rely on global pooling, it is commonly used in channel attention to encode spatial information. However, global pooling compresses spatial information into a single channel descriptor, making it difficult to retain positional cues, which are essential for capturing spatial structures in visual tasks. In contrast, the CA mechanism enhances spatial feature recognition by embedding precise positional information into channel attention. The structure of the CA module is illustrated in Fig 5.

**Fig 5:**
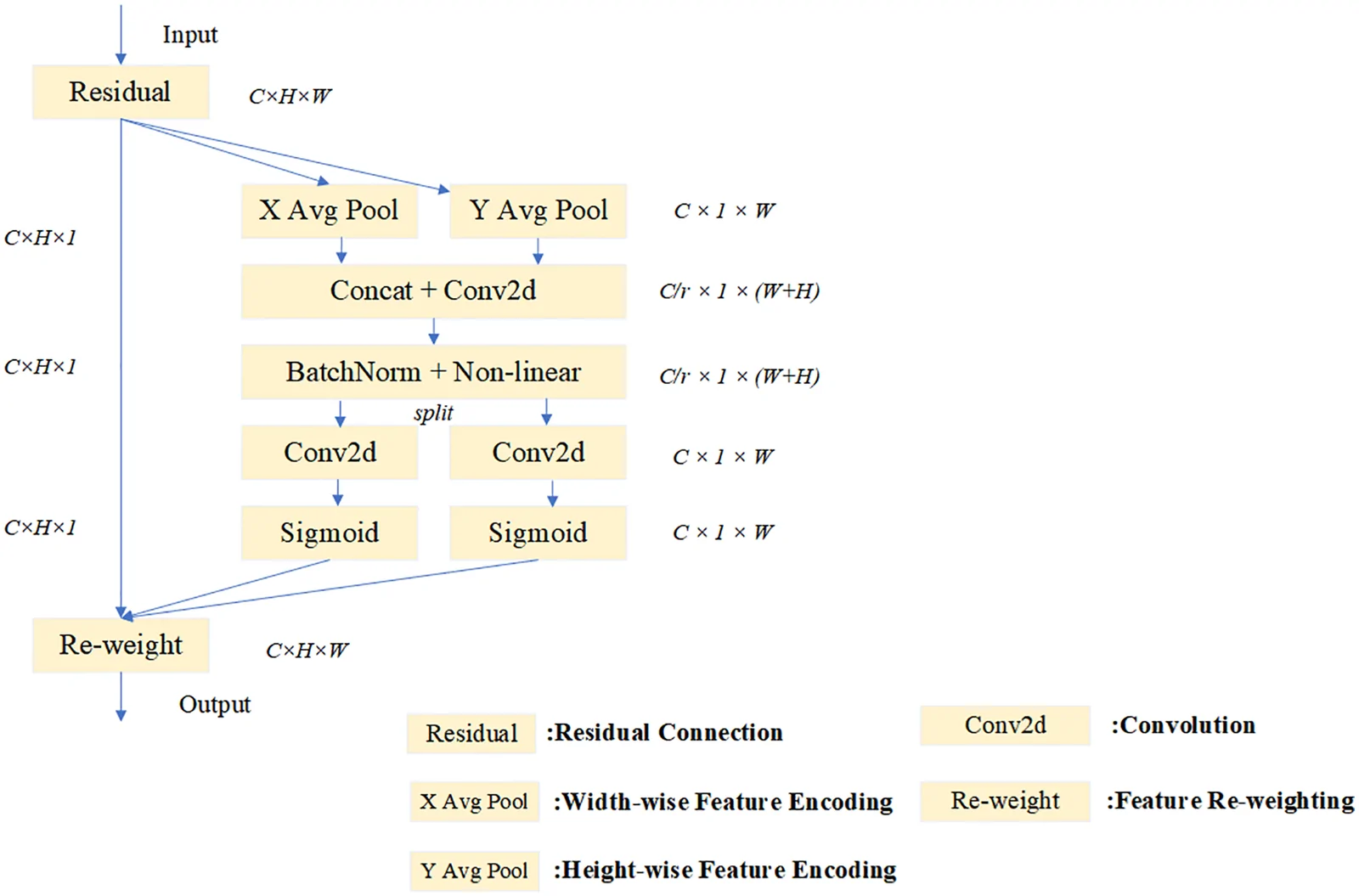
Coordinate attention block.

To enable attention blocks to more effectively capture long-range spatial dependencies with accurate positional information, the CA mechanism splits the computational process of channel attention into two independent directional components using a 1D feature encoding operation. Specifically, given an input $X=[x_{\text{1}},x_{\text{2}},...,x_{C}]\in R^{C\times H\times W}$, two pooling kernels with spatial extents of (H, 1) and (1, W) are applied to encode each channel along the horizontal and vertical axes, respectively. Thus, the output of the a-th channel with height h is shown in Equation (1):


$$\begin{array}{c} d_{a}^{h}\left(h\right)=\frac{\text{1}}{W}\sum_{0\leq i<w}x_{a}\left(h,i\right) \end{array}$$ (1)

Similarly, the output of the a-th channel of width w is shown in Equation (2):


$$\begin{array}{c} d_{a}^{w}\left(w\right)=\frac{\text{1}}{H}\sum_{0\leq j<H}x_{a}\left(j,w\right) \end{array}$$ (2)

These two transformations enable the attention blocks to effectively capture long-range dependencies along one spatial axis while preserving precise positional information along the other, thereby enhancing the network’s ability to accurately localize objects of interest. Subsequently, the aggregated feature maps generated by Equation 1 and Equation 2 are first connected and then passed through the shared 1 × 1 convolutional transform function $F_{\text{1}}$ to produce the final combined representation shown in Equation (3):


$$\begin{array}{c} f=\delta \left(F_{\text{1}}\left(\left[d^{h},d^{w}\right]\right)\right) \end{array}$$ (3)

where [-,-]denotes the concatenation operation along the spatial dimension, δ is a nonlinear activation function, and $\in R^{C/r\times (H+W)}$ is an intermediate feature map used to encode spatial information in both the horizontal and vertical directions. Here, r is the reduction ratio for controlling the block size. The feature map f is then partitioned into two independent tensors, $f^{h}\in {\text{R}}^{C/r\times H}$ and $f^{w}\in {\text{R}}^{C/r\times W}$, along the spatial dimension. Next, two 1 × 1 convolutional transforms $F_{h}$ and $F_{w}$ are utilized to transform ${\text{f}}^{h}$ and ${\text{f}}^{w}$, respectively, into tensors with the same number of channels as the input X to obtain:


$$\begin{array}{c} p^{h}=\sigma \left(F_{h}\left({\text{f}}^{h}\right)\right) \end{array}$$ (4)

$$\begin{array}{c} p^{w}=\sigma \left(F_{w}\left({\text{f}}^{w}\right)\right) \end{array}$$ (5)

where σ denotes the sigmoid function, and the outputs $p^{h}$ and $p^{w}$ are applied as attention weights along the corresponding spatial dimensions, respectively. Finally, the output of the coordinate attention module, denoted as Y, is formulated as follows:


$$\begin{array}{c} y_{a}\left(i,j\right)=x\left(i,j\right)\times p_{a}^{h}\left(i\right)\times p_{a}^{w}\left(j\right) \end{array}$$ (6)

This process enables the model to dynamically adjust attention weights based on the importance of different regions, thereby enhancing its focus on salient feature areas.

Compared with traditional attention methods such as Squeeze-and-Excitation (SE) and Convolutional Block Attention Module (CBAM), which either ignore positional information or only capture local spatial dependencies, the CA mechanism provides a more efficient and comprehensive approach to capture inter-channel relationships and remote dependencies with precise positional information, improving spatial feature discrimination and thus helping the model to better classify brain tumor images.

Subsequently, Conv2 is employed to reduce the computational complexity of the model.

Finally residual connections were used to ensure that network information was not lost.

#### 2.3.2. CI block.

The structure of the CI block is shown in Fig 6, which is designed to efficiently downsample the input data. Downsampling plays a crucial role in neural network architectures [33] to reduce the number of parameters and improve the generalization of the model. However, during the downsampling process, the model discards a portion of the data, which may lead to the loss of critical information. Therefore, convolution and pooling are proposed to complement each other to solve this problem.

**Fig 6:**
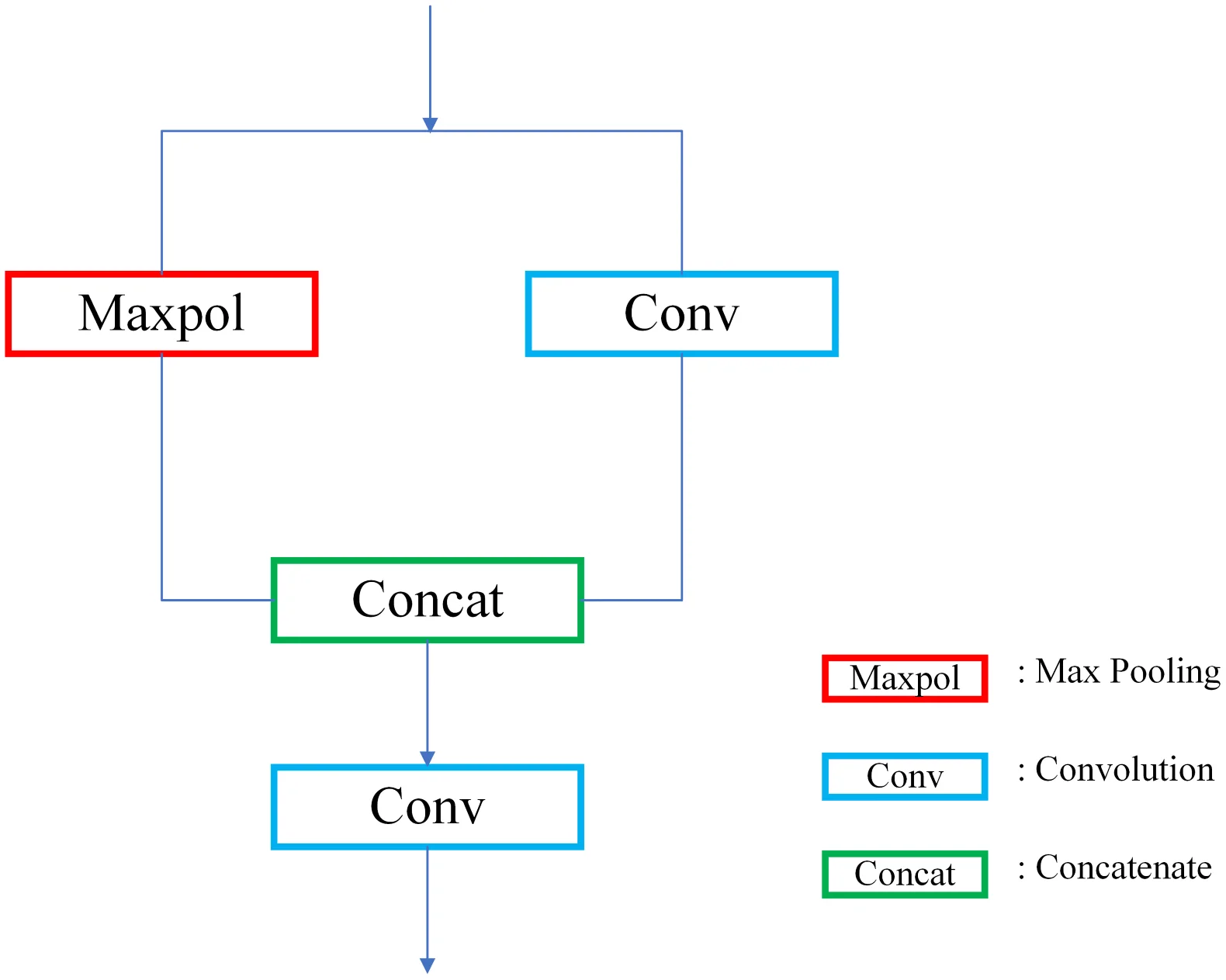
CI block.

The CI block is characterized by the complementary use of convolution and pooling. The convolutional layer captures detailed information such as edges in images through the sliding operation of convolution kernels on the input data. Conversely, the pooling layer diminishes the dimensionality of the feature map generated by the convolutional layer through max pooling, which effectively shrinks the feature map and mitigates data redundancy. This complementarity enables the network to fully extract useful information from images while also reducing computational complexity and storage requirements. Additionally, the complementarity is evident in the expansion of receptive fields. Due to the limited size of convolution kernels, the input region covered by a single convolutional layer (i.e., the receptive field) is also limited. However, the pooling layer enlarges the receptive fields of subsequent convolutional layers through downsampling, enabling the network to capture more global information.

### 2.4. Training CNNs

The training of a convolutional neural network comprises both forward and backward propagation stages. It commences at the input layer and progressively propagates information forward through the network structure. Subsequently, loss values are back-propagated layer by layer from the output layer to the first layer of the network. During this process, the i-th neuron in layer l receives input from neuron j in the preceding layer (i.e., layer l-1) and computes its output value through a series of computations. This process of calculation is handled by weighted summation and activation function, the weighted summation is shown in Equation (7).


$$\begin{array}{c} In=\sum_{j=1}^{n}W_{ij}^{\text{1}}x_{j}+b_{i} \end{array}$$ (7)

where,$\;W_{ij}^{\text{1}}x_{j}\;$represents the weights, and${\;b}_{i\;}$denotes the bias term. Initially, we compute the weighted sum (In) of the variables to obtain an intermediate result. Then, the intermediate value undergoes processing through an activation function, with the Swish and ReLU activation functions, defined by Equations (8) and (9), respectively.


$$\begin{array}{c} S_{i}^{\text{1}}=In_{i}^{\text{1}}\times sigmoid\left(\beta In_{i}^{\text{1}}\right) \end{array}$$ (8)

$$\begin{array}{c} R_{i}^{l}=max\left(0,In_{i}^{l}\right) \end{array}$$ (9)

where, $S_{i}^{\text{1}}\;$represents the output value after processing by the Swish activation function, and β is a constant parameter. Similarly, $R_{i}^{l}$ denotes the output obtained by the ReLU activation function. The output values of each neuron in both the convolutional and fully connected layers are then precisely computed by using equations (8) and (9). The use of activation functions for the weighted values here is to convert the input values into nonlinear outputs so that the proposed model can learn and express complex features.

The primary objective of the cross-entropy loss function is to precisely evaluate the model’s prediction error. This is achieved by comparing the predicted probability distribution generated by the model with the true labeling distribution, as depicted in Equation (10). Utilizing the back-propagation mechanism, the function optimizes the model parameters, consequently enhancing the accuracy of prediction outcomes. This approach ensures both the objectivity of assessment and the reliability of model predictions.


$$\begin{array}{c} L=\frac{-1}{m}\sum_{i}^{m}ln\left(p\left(\frac{y_{i}}{x_{i}}\right)\right) \end{array}$$ (10)

During back propagation, the proposed model updates the weights based on the gradient of the loss function. The model weights are updated according to Equation (11).


$$\begin{array}{c} \begin{array}{c} \gamma^{t}=\gamma^{\left[\frac{tN}{M}\right]}\\ V^{t+1}=\mu V_{\text{1}}^{t}-\gamma^{t}\alpha_{\text{1}}\frac{\partial C}{\partial W}\\ W_{i}^{t+1}=W_{\text{1}}^{t}+V_{t}^{t+1} \end{array} \end{array}$$ (11)

where, the parameters $\;\alpha_{\text{1}}$, $\gamma^{t}$, and  μ  significantly influence the learning algorithm’s performance throughout the iterative process. Specifically, $\alpha_{\text{1}}$, representing the learning rate of layer l, directly influences the weight update magnitude, ensuring model convergence during training. Meanwhile, $\gamma^{t}$ governs the learning schedule, optimizing algorithm convergence by controlling the reduction rate of initial values. Furthermore, the interplay between the learning rate and  μ  effectively characterizes the impact of previous weight updates on the current iteration, enhancing model generalization ability. The proposed model is optimized using this method to gradually reduce the error.

## 3. Results

### 3.1. System implementation and evaluation metrics

The experiments were conducted on a computer equipped with a 3.50 GHz CPU, 64.0 GB RAM, and an NVIDIA GeForce RTX 4070 Ti. All experiments were conducted in Python 3.10 environment. PyTorch 2.1.1 and TorchVision 0.16.1 are used to implement the proposed model.

The Adam optimizer was utilized for training with an initial learning rate of 0.001 for 60 iterations. The learning rate decayed by a factor of 0.1 every 10 iterations. The batch size was set to 32 samples. The value of network training hyperparameters are provided in Table 2 The performance of the proposed method was evaluated using accuracy, sensitivity, precision, specificity, and F1 score metrics, calculated as described below [34].

**Table 2:** Training hyperparameter values for the proposed model.

Hyper-parameter	Value
Optimizer	Adam
Initial learning rate	0.001
Number of epochs	60
Learning rate decays	0.1
Mini-batch size	32


$$\begin{array}{c} A\text{ccuracy}=\frac{TP+TN}{TP+TN\text{+}FP\text{+}FN} \end{array}$$ (12)

$$\begin{array}{c} Sensitivity=\frac{TP}{TP+FN} \end{array}$$ (13)

$$\begin{array}{c} Precision=\frac{TP}{TP+FP} \end{array}$$ (14)

$$\begin{array}{c} F1-Score=\frac{2\times Precision\times Recall}{Precision+Recall} \end{array}$$ (15)

True Positives (TP) are referred to as the instances that are accurately identified as positive among all the tumor cases predicted as positive. Likewise, True Negatives (TN) are denoted as the cases that were indeed negative among those predicted as negative. Conversely, False Negatives (FN) indicate cases that were wrongly predicted as negative when they were actually positive. False Positives (FP) signify cases that were predicted as positive but were actually negative.

To ensure the robustness of our performance evaluation, we performed 10-fold cross-validation. The experimental results are presented in Table 3. The dataset was randomly partitioned into ten subsets of approximately equal size. In each fold, one subset was used as the validation set, and the remaining nine were used for training. This process was repeated ten times, resulting in ten accuracy scores. The mean accuracy of 98.04% and standard deviation of 0.98%, as presented in Table 3, demonstrate the stability of the proposed model.

**Table 3:** 10-fold cross-validation results on the Chen dataset.

Fold	Accuracy (%)
1	97.97
2	98.37
3	98.37
4	98.78
5	96.73
6	98.37
7	98.37
8	99.59
9	95.92
10	97.96
**Mean ± Std**	**98.04 ± 0.98**

The main steps of the brain tumor classification experiment include the training phase and the testing phase. The process unfolds as follows.

1. The magnetic resonance imaging dataset is partitioned into mutually exclusive training and testing subsets.
2. Augmentation techniques such as random rotation and horizontal flipping are applied to the training dataset to augment the image count and mitigate overfitting.
3. A modular network is constructed, and its weights are initialized randomly using the default method.
4. The network undergoes training with specified hyperparameter values.
5. Upon completion of training, the model is saved to disk.
6. Finally, model evaluation is conducted using various metrics on the designated test dataset.

### 3.2. The ablation study

To assess the impact of each module on the proposed model, a series of experiments were conducted and the effect of combining different blocks was detailed in Table 4. Firstly, the basic model Baseline was used. Then, the ConvDAtt module was integrated into the basic model Baseline, forming the Baseline-ConvDAtt module. Subsequently, the CI module was incorporated, resulting in the Baseline-ConvDAtt-CI module. Finally, by combining the SPP module with the Baseline-ConvDAtt-CI module, the Baseline-ConvDAtt-CI-SPP module was obtained.

**Table 4:** Detailed metrics values of the proposed model on the Chen dataset.

Method	Tumor type	Pre. (%)	Rec. (%)	F1. (%)	Acc. (%)	Training time(min)	Inference time(s)
Baseline	Glioma	89.27	81.75	85.35	**83.82**	78.25	4
	Meningioma	65.81	72.34	68.92			
	Pituitary	90.82	95.70	93.19			
	**Average**	81.97	83.26	82.49			
Baseline- ConvDAtt	Glioma	97.91	98.60	98.25	**97.39**	359.2	6
	Meningioma	93.71	95.04	94.37			
	Pituitary	99.45	97.31	98.37			
	**Average**	**97.02**	**96.98**	**97.00**			
Baseline- ConvDAtt-CI	Glioma	98.26	98.95	98.60	**98.37**	35.77	4
	Pituitary	99.46	98.39	98.92			
	Meningioma	97.16	97.16	97.16			
	**Average**	**98.29**	**98.17**	**98.23**			
Baseline- ConvDAtt-CI-SPP	Glioma	98.95	98.95	98.95	**98.69**	78.98	5
	Meningioma	96.53	98.58	97.54			
	Pituitary	100.00	98.39	99.19			
	**Average**	**98.49**	**98.64**	**98.56**			

Table 4 summarizes the performance variations resulting from the progressive integration of different modules. The baseline model achieves an accuracy of 83.82%. After incorporating the ConvDAtt block into the baseline model, the accuracy increases significantly to 97.39%, indicating that ConvDAtt effectively enhances discriminative feature extraction. Subsequently, the incorporation of the CI block increases the accuracy to 98.37%. Finally, the addition of the SPP block leads to a final accuracy of 98.69%, achieving the overall best performance. In addition to classification performance, Table 4 also provides computational efficiency indicators. The proposed model requires 78.98 minutes for training and 5 seconds for inference, indicating acceptable computational efficiency. Although incorporating the ConvDAtt block substantially improves classification accuracy, it also introduces additional computational overhead, resulting in increased training time. After the introduction of the CI module, the training time was reduced from 359.2 minutes to 35.77 minutes. The training time was significantly shortened while maintaining a competitive accuracy rate, indicating that the CI module has improved computational efficiency without sacrificing performance. The final Baseline-ConvDAtt-CI-SPP model achieves a favorable balance between classification performance and computational cost. Overall, the proposed modules play complementary roles in the network. The ConvDAtt block mainly enhances discriminative feature extraction, the CI block focuses on information preservation and computational efficiency during downsampling, and the SPP block improves multi-scale feature representation.

The analysis shows that the accuracy of the model on the basical model Baseline is 83.82%. Then, by integrating the ConvDAtt block into the baseline model, the accuracy of this model reaches 97.39%. Subsequently, after adding the CI block, the accuracy of the model rises to 98.37%. Finally, by adding the SPP block, the accuracy of the model is further increased to 98.69%. This underscores the distinctive benefits of utilizing the ConvDAtt, CI, and SPP blocks in neural network architectures. Meanwhile, some indicators of computational efficiency are given in Table 4. For example, the training time of the proposed model is 78.98 minutes, the inference time is 5 seconds. These metrics indicate that efficient computational performance is exhibited by the proposed model. Moreover, by incorporating the ConvDAtt module into the baseline model, the accuracy of the resulting Baseline-ConvDAtt model increases significantly, whereas the training time grows by a factor of several times. Subsequently, with the incorporation of the CI module, the training time is significantly reduced without compromising accuracy. The proposed final model, termed Baseline-ConvDAtt-CI-SPP, reduces computational time while preserving high accuracy, thereby demonstrating its high efficiency.

To rigorously assess the statistical significance of the improvements brought by each proposed module, we conducted paired t-tests based on five independent runs using different random seeds on the Chen dataset. As shown in Table 5, the average accuracy rate of the proposed model is 24.68% higher than that of the baseline model. The paired t-test yielded a t-value of 32.94 and p < 0.001, confirming that the overall improvement was statistically significant. The introduction of the ConvDAtt block brought about the most significant gain, with an accuracy improvement of 23.79% and p < 0.001, highlighting its crucial role in enhancing feature extraction and training stability. Although the subsequent integration of the CI and SPP blocks resulted in more modest numerical improvements of 0.76% and 0.13%, respectively, these differences did not reach statistical significance (p > 0.05). Nevertheless, these modules contribute to improved model parameter efficiency and reduced computational cost, benefits that are reflected in enhanced stability and overall performance. Overall, the statistical analysis confirms that the proposed method achieves significant and reliable improvements compared to the baseline.

**Table 5:** Paired t-test results.

Comparison group	Average improvement in Acc (%)	t-value	P-value	Is it significant
Baseline vs. Baseline+ConvDAtt	+23.79	44.69	<0.001	Yes
Baseline+ConvDAtt vs. + CI	+0.76	1.86	0.136	No
Baseline+ConvDAtt + CI vs. + SPP	+0.13	0.26	0.807	No
**Baseline vs. Proposed Model**	**+24.68**	**32.94**	**<0.001**	**Yes**

The results presented in Table 6 demonstrate that the proposed model exhibits favorable efficiency with respect to parameter scale, computational complexity, and model size. Specifically, the final model (Baseline-ConvDAtt-CI-SPP) contains 15.29 M parameters, requires 2.85 G FLOPs, and occupies a model size of 58.31 MiB. Furthermore, when transitioning from Baseline-ConvDAtt to Baseline-ConvDAtt-CI, the FLOPs decrease significantly from 97.14 G to 2.40 G, while classification accuracy is further improved to 98.37%. This observation strongly confirms that the CI module effectively enhances feature representation capability while simultaneously reducing computational cost.

**Table 6:** Model size and cost indicators.

Method	Parameter quantity(M)	Flops(G)	Model size (MiB)	Accuracy (%)
Baseline	0.01	0.73	0.06	83.82
Baseline- ConvDAtt	2	97.14	7.62	97.39
Baseline- ConvDAtt-CI	5.85	2.40	22.3	98.37
Baseline- ConvDAtt-CI-SPP	15.29	2.85	58.31	98.69

Considering that low-quality images may adversely affect the performance of the proposed model, we conducted a series of ablation experiments to evaluate its robustness. The detailed results are presented in Table 7.

**Table 7:** Metric values of the proposed model post-gaussian noise processing on the Chen dataset.

Method	Tumor type	Pre. (%)	Rec. (%)	F1. (%)	Acc. (%)
Baseline	Glioma	48.80	99.65	65.51	51.14
	Meningioma	0.000	0.000	0.000	
	Pituitary	96.67	15.59	26.85	
	**Average**	48.49	38.41	30.79	
Baseline -ConvDAtt	Glioma	89.71	55.09	68.26	63.24
	Meningioma	97.78	31.21	47.31	
	Pituitary	47.45	1.000	64.36	
	**Average**	78.31	62.10	59.98	
Baseline -ConvDAtt-CI	Glioma	98.12	73.33	83.94	83.99
	Pituitary	65.24	86.52	74.39	
	Meningioma	86.32	98.39	91.96	
	**Average**	83.23	86.08	86.76	
Baseline -ConvDAtt-CI-SPP	Glioma	97.89	97.54	97.72	96.90
	Meningioma	91.22	95.54	93.43	
	Pituitary	1.000	96.77	98.36	
	**Average**	96.37	96.62	96.50	

Gaussian noise is a common type of random noise. In this section, the model’s performance is evaluated by simulating low-quality images through the addition of Gaussian noise. Table 7 presents the ablation results and the performance of the proposed model under low-quality image conditions. As shown in Table 7, the accuracy of the baseline model decreases significantly after introducing Gaussian noise. After incorporating the ConvDAtt and CI modules, the model gradually recovers its discriminative capability. With the further addition of the SPP module, the accuracy increases to 96.90%, demonstrating improved robustness under degraded image conditions. Furthermore, the effectiveness of the SPP module is more evident under degraded image conditions. The robustness experiment demonstrates that the complete model maintains higher performance under noisy inputs, indicating that multi-scale feature aggregation helps improve feature representation in challenging scenarios. This indicates that the SPP module enhances robustness against image quality degradation by strengthening multi-scale feature representation.

To assess the interactions between convolution and pooling layers in the CI block by separately excluding convolution or pooling layers from the CI block. Table 8 provides detailed indicators of the model after removing the convolutional or pooled layer from the CI block.

**Table 8:** Detailed metrics of the model after removing convolution or pooling from the CI block on the Chen dataset.

Method	Tumor type	Precision (%)	Recall (%)	F1-score (%)	Accuracy (%)
Single pooling	Glioma	94.93	98.60	96.73	96.57
	Meningioma	95.49	90.07	92.70	
	Pituitary	100.00	98.39	99.19	
	Average	96.81	95.69	96.21	
Single convolution	Glioma	96.88	97.89	97.38	97.39
	Meningioma	96.38	94.33	95.34	
	Pituitary	98.92	98.92	98.92	
	Average	97.39	97.05	97.21	

From Table 8, it can be observed that upon eliminating the convolutional layer from the CI block, the model classification accuracy was 96.57%. Similarly, upon removing the pooling layer, the model classification accuracy was 97.39%. However, the classification accuracy for our proposed completed model attained 98.69%. In conclusion, the synergistic integration of convolution and pooling within the CI block is advantageous in enhancing the model’s overall performance.

### 3.3. Impact of data augmentation

Due to the restricted sample size in the benchmark dataset, overfitting may occur during the training of the proposed model. To mitigate this issue, a data augmentation approach was utilized to increase sample diversity and enhance system performance [35]. Each image was applied with random rotations within a ± 15 degrees range and horizontal flips were conducted with a 50% probability. These adjustments aim to comprehensively capture data features, thereby optimizing model training.

In order to thoroughly validate the data enhancement method adopted in this experiment, the Chen dataset and the Kaggle dataset were used for the experiment. The experimental results are shown in Fig 7. As can be observed from Fig 7, the data augmentation technique significantly improves the model performance. Specifically, the accuracy has been increased by 3.1% on the Chen dataset and 2.44% on the Kaggle dataset.

**Fig 7:**
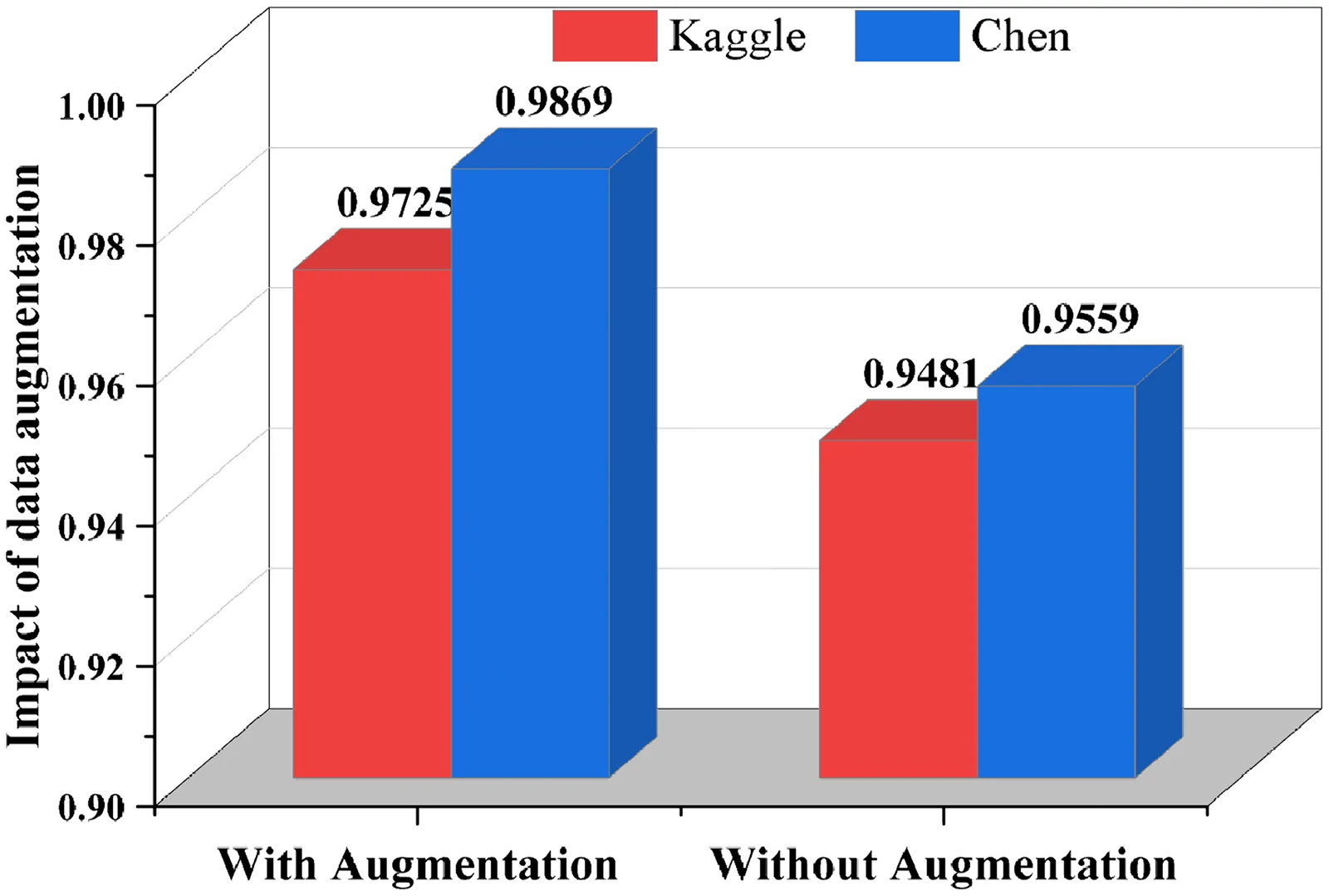
Impact of data augmentation.

## 4. Discussion

A confusion matrix [36] is a specific tabular layout that is primarily used to evaluate the performance of a classification model to clearly show how well the classification model predicts actual samples. The confusion matrices of the proposed model on the Chen and Kaggle datasets are shown in Fig 8 and Fig 9, respectively. Equations (12) to (15) are utilized to compute the specific values of the model’s classification outcomes from the confusion matrixes. The symbols G, M, P, and N designate distinct types of brain tumors, with G standing for glioma, M for meningioma, P for pituitary tumor, and N representing no tumor. From the confusion matrix on the Chen dataset, it can be calculated that the precision of glioma classification reached 98.95%, the precision of meningioma classification reached 96.53%, and the precision of pituitary tumor classification reached 100%. The results indicate that the proposed model has a good classification ability for various types of brain tumors.

**Fig 8:**
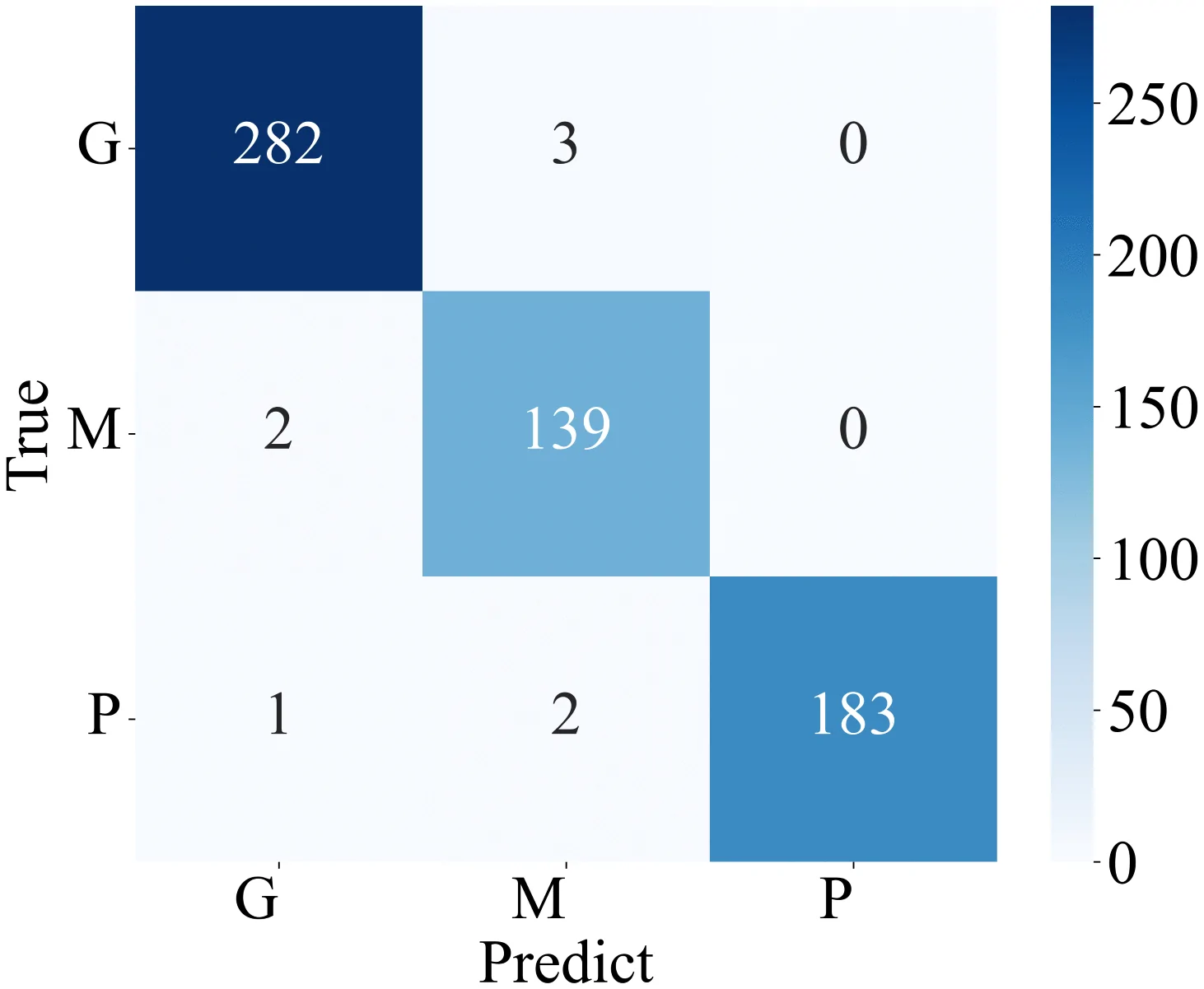
The confusion matrix of the proposed model on the Chen dataset.

**Fig 9:**
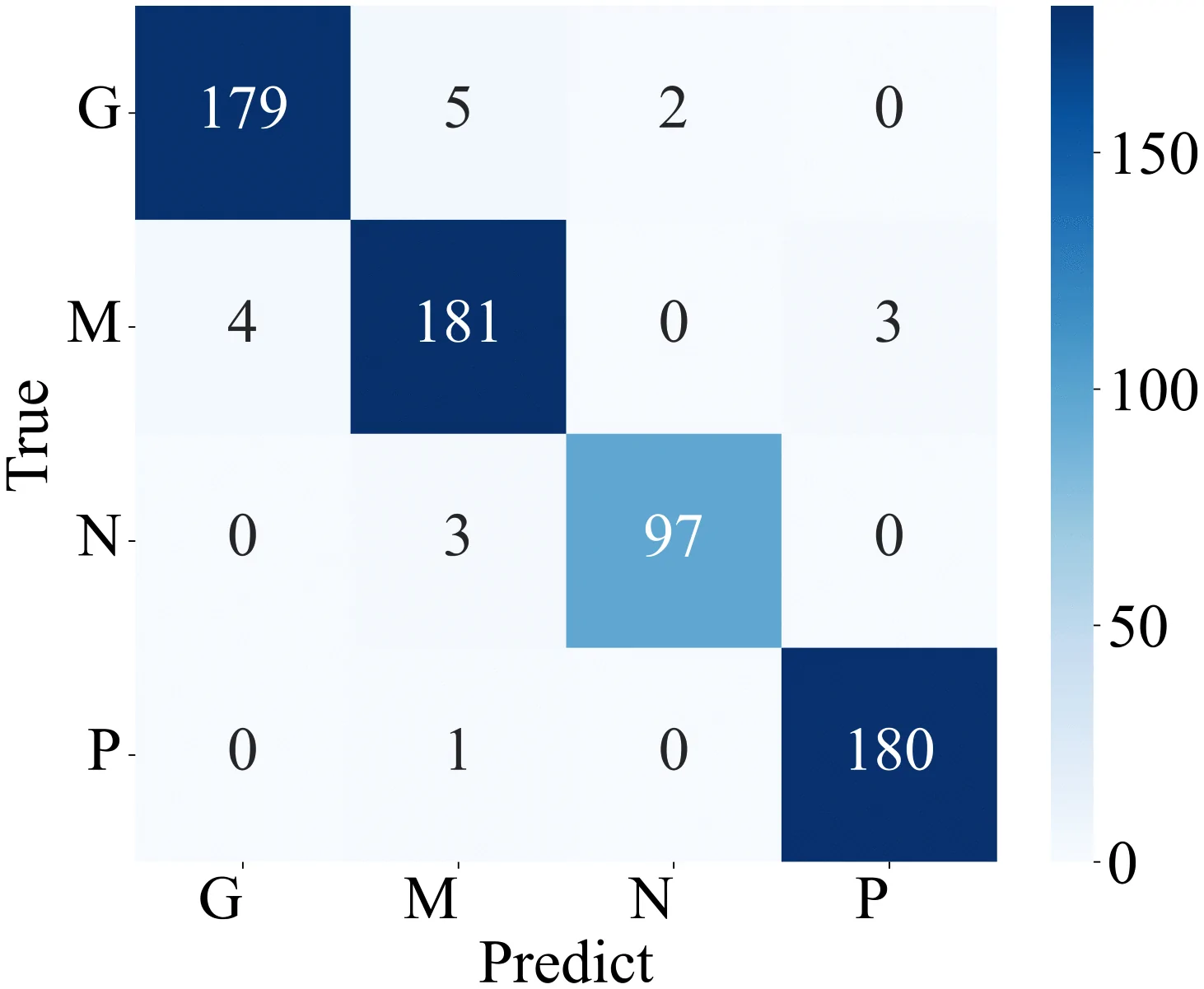
The confusion matrix of the proposed model on the Kaggle dataset.

To offer a comprehensive assessment of the proposed model’s performance spanning various classes, Table 9 and Table 10 exhibit the specific precision, recall, and F1-score for each individual class on the Chen and Kaggle datasets, respectively.

**Table 9:** The performance index of the proposed model on the Chen dataset.

Class	Precision (%)	Recall (%)	F1-score (%)
Glioma	98.95	98.95	98.95
Meningioma	96.53	98.58	97.54
Pituitary	100.00	98.39	99.19

**Table 10:** The Performance indicators of the proposed model on the Kaggle dataset.

Class	Precision (%)	Recall (%)	F1-score (%)
Glioma	97.81	96.24	97.02
Meningioma	95.26	96.28	95.7
Pituitary	97.98	97.00	97.9
No Tumor	98.36	99.45	98.0

These detailed metrics highlight the robust performance of our proposed model across different brain tumor types and the normal class. The ability of the model to accurately classify each class is demonstrated by the high precision, recall, and F1-scores, which further validates the effectiveness of the ConvDAtt, CI, and SPP blocks in enhancing the overall performance. Furthermore, we calculated the accuracy for each image and subsequently computed the standard deviation. The standard deviation on the Chen dataset was 0.1136, while that on the Kaggle dataset was 0.1635. The relatively small standard deviations on both datasets suggest that the model is stable and reliable.

The systematic comparison between the proposed model and previous state-of-the-art models using the same dataset is exhaustively demonstrated in Table 11. The results of the comparative analysis clearly reveal that the proposed model demonstrates superiority over these state-of-the-art approaches on both the Chen and Kaggle datasets. Specifically, the proposed model achieves an accuracy of 98.69% on the Chen dataset and 97.25% on the Kaggle dataset. Particularly, the accuracy of the proposed model on the Chen dataset is improved by 0.27% compared to the best model, Enhanced AlexNet with ASA [37], while on the Kaggle dataset, it is improved by 0.25% compared to the best model, EfficientNet-B7++ [38]. In addition, the proposed model outperforms previous state-of-the-art models in terms of F1-score, Precision, and Recall metrics on both the Chen and Kaggle datasets. These performance improvements are mainly attributed to the in-depth improvements and innovations in our methodology, especially in the areas of data preprocessing, feature extraction, and the handling of complementary relationships between pooling and convolutional layers. These improvements greatly improve the accuracy of the model, thus making the proposed model stand out in comparison with other state-of-the-art models.

**Table 11:** Comparison of our proposed model with previous models.

Reference	Year	Method	Dataset	Acc. (%)	F1 (%)	Pre. (%)	Rec.(%)
Bartosz Sterniczuk et al. [39]	2024	F1-WMVE	Chen	94.14	–	–	–
Gergo Bogacsovics et al. [40]	2024	MobileNetv2	Chen	92.10	92.10	92.50	92.00
Suliman Farhan et al. [41]	2025	PRCnet	Chen Kaggle	96.51 92.70	96.49 92.66	96.50 92.75	96.51 92.70
Ahmad Ishaq et al. [38]	2025	EfficientNet-B7++	Chen Kaggle	96.00 97.00	– 91.00	– 91.00	– 92.00
Afnan M. Alhassan et al. [42]	2025	IV3TM	Chen	97.59	97.58	97.98	97.20
Guo et al. [43]	2024	ResNet18 + ShallowNet + SVM	Chen	97.50	–	–	–
Dutta TK et al. [44]	2024	ARM-Net	Chen	97.11	96.87	96.66	97.97
Shynar Akhmetzhanova et al. [45]	2024	Prototypical Networks	Chen	69.35	65.34	66.77	64.66
Sebastián Felipe Álvarez et al. [46]	2024	ResNet50 (Based on the confusion matrix)	Chen	97.00	–	–	–
Balasuburamani et al. [37]	2025	Enhanced AlexNet with ASA	Chen	98.42	96.03	97.30	97.62
Proposed model	2025	ConvDAtt + CI + SPP	Chen Kaggle	98.69 97.25	98.56 97.30	98.49 97.35	98.64 97.24

Table 12 presents the parameter count and accuracy of models evaluated on the Chen dataset over the past two years. As can be observed, the proposed model achieves a parameter count of 15.29M and achieves an accuracy of 98.69%. Regarding the Enhanced AlexNet with ASA [37]model, which achieves an accuracy closest to that of the proposed model, its parameter count ranges from 60M to 70M, which is approximately three to four times that of the proposed model. For models with fewer parameters than the proposed model, namely MobileNetV2 [40] EfficientNet-B7++ [38], and Prototypical Networks [45], the reported accuracies are 92.10%, 95.00%, and 69.35%, respectively, all of which are considerably lower than the accuracy of the proposed model. In addition, the parameter counts for these three models are 11.1M, 8.38M, and 11.7M, which are only one to two times smaller than that of the proposed model.

**Table 12:** Parameter details of comparative models on the Chen dataset.

Reference	Method	Accuracy (%)	Parameter quantity(M)
Bartosz Sterniczuk et al. [39]	F1-WMVE	94.14	260.32
Gergo Bogacsovics et al. [40]	MobileNetv2	92.10	11.1
Suliman Farhan et al. [41]	PRCnet	96.51	21.41
Ahmad Ishaq et al. [38]	EfficientNet-B7++	95.00	8.38
Afnan M. Alhassan et al. [42]	IV3TM	97.59	24
Guo et al. [43]	ResNet18 + ShallowNet + SVM	97.50	–
Dutta TK et al. [44]	ARM-Net	97.11	113
Shynar Akhmetzhanova et al. [45]	Prototypical Networks	69.35	11.7
Sebastián Felipe Álvarez et al. [46]	ResNet50 (Based on the confusion matrix)	97.00	24.64
Balasuburamani et al. [37]	Enhanced AlexNet with ASA	98.42	60-70
Proposed model	ConvDAtt + CI + SPP	98.69	15.29

Fig 10 and Fig 11 depict the Receiver Operating Characteristic (ROC) curves of the proposed model on the Chen dataset and the Kaggle dataset, respectively. The Area Under the Curve (AUC) is fundamentally assessed as a metric for evaluating the effectiveness of the model in distinguishing between various tumor types. High or low AUC values directly reflect classifier performance. On the Chen dataset, AUC values of 0.9970, 0.9975, and 0.9994 are achieved by the proposed model for glioma, meningioma, and pituitary tumor, respectively. Similarly, on the Kaggle dataset, AUC values of 0.9837, 0.9904, 0.9936, and 0.9988 are attained by the proposed model for the four categories of glioma, meningioma, pituitary tumor, and no tumor, respectively. These results comprehensively illustrate the excellent performance of the model in classifying various tumor types.

**Fig 10:**
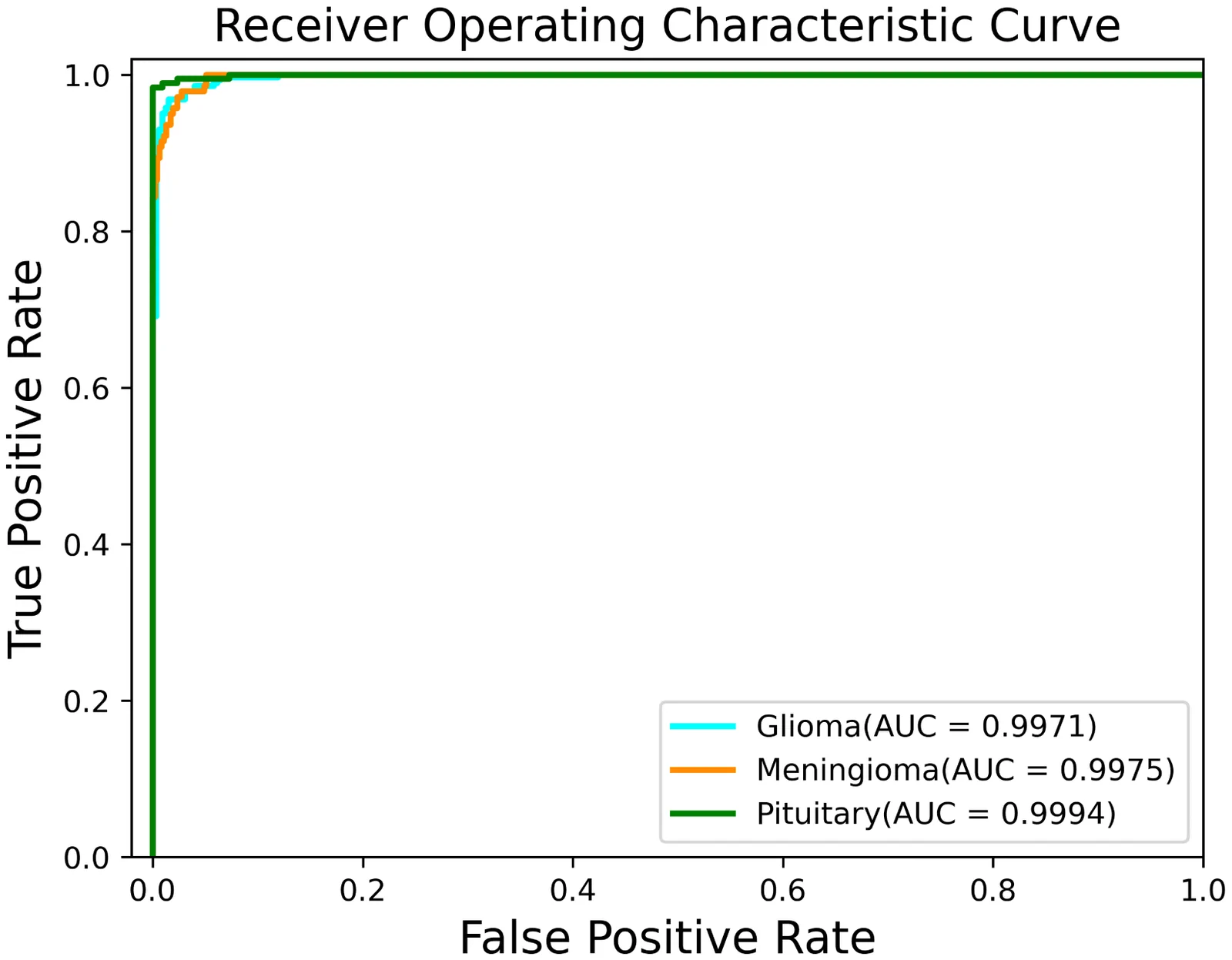
The ROC curve of the proposed model on the Chen dataset.

**Fig 11:**
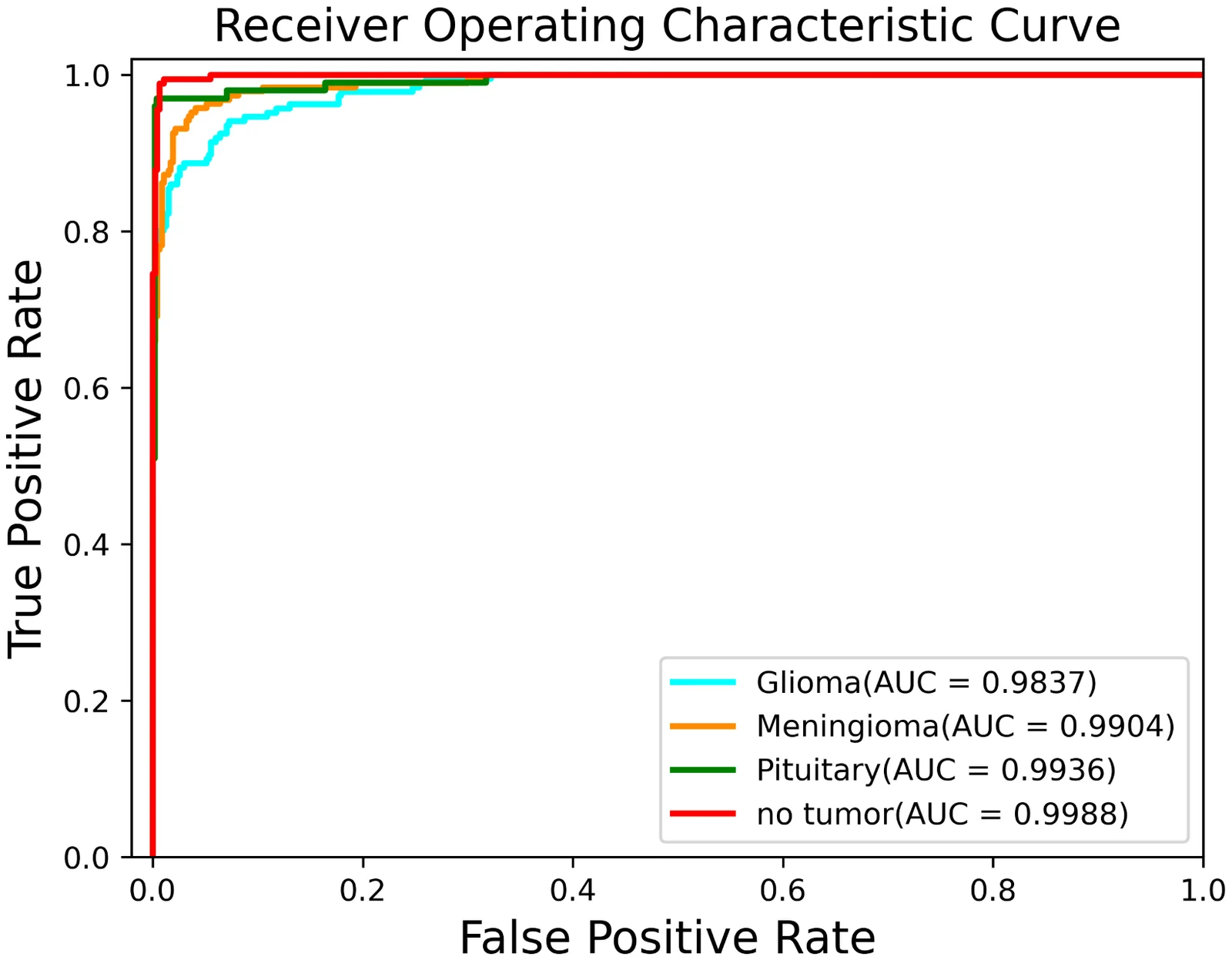
The ROC curve of the proposed model on the Kaggle dataset.

Fig 12 and Fig 13 demonstrate the excellent performance of the proposed model through visualization. As can be seen in Fig 12 and Fig 13, the samples within each class are more compactly distributed, and the distances between classes are increased.

**Fig 12:**
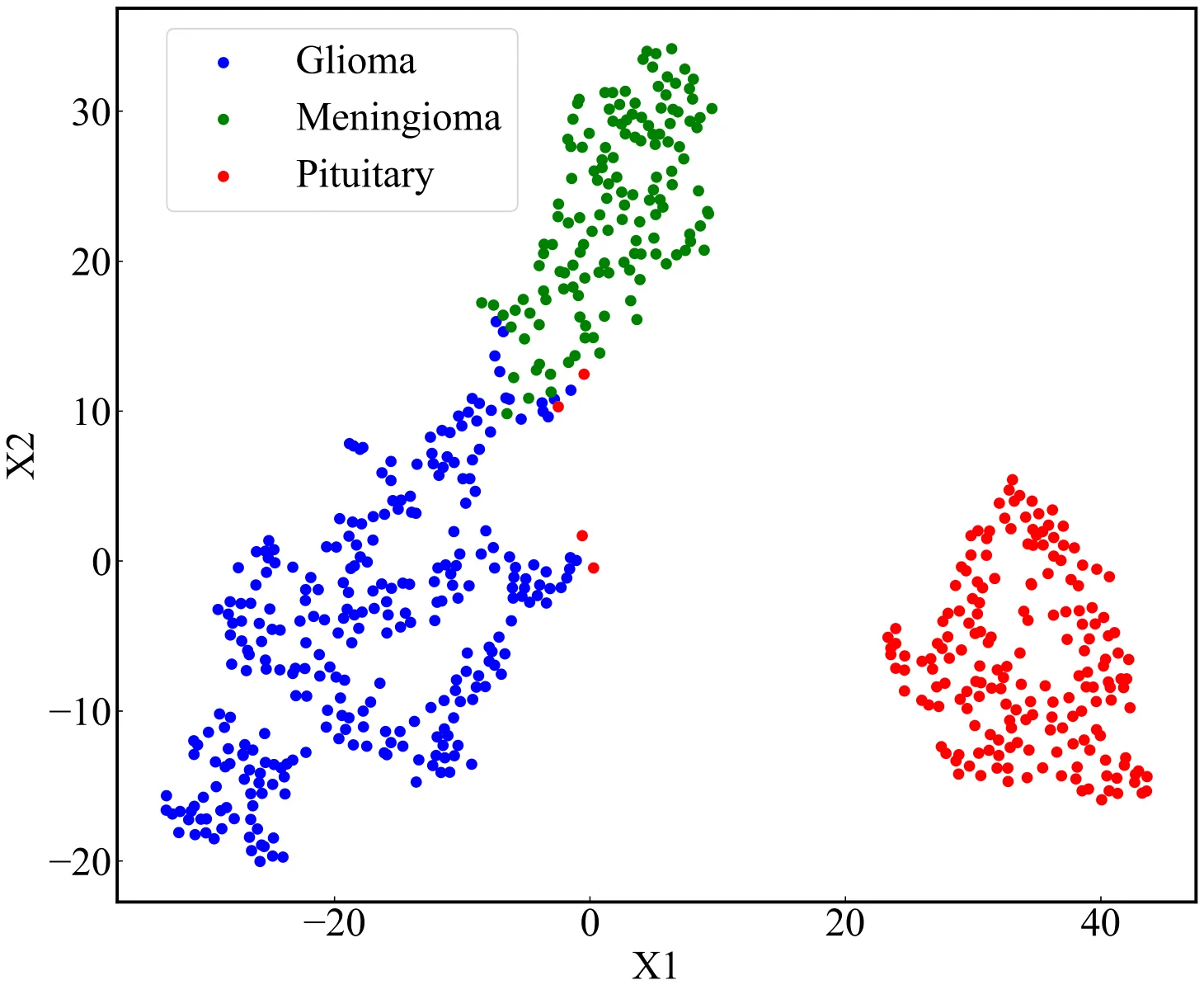
The scatter plot of the proposed model on the Chen dataset.

**Fig 13:**
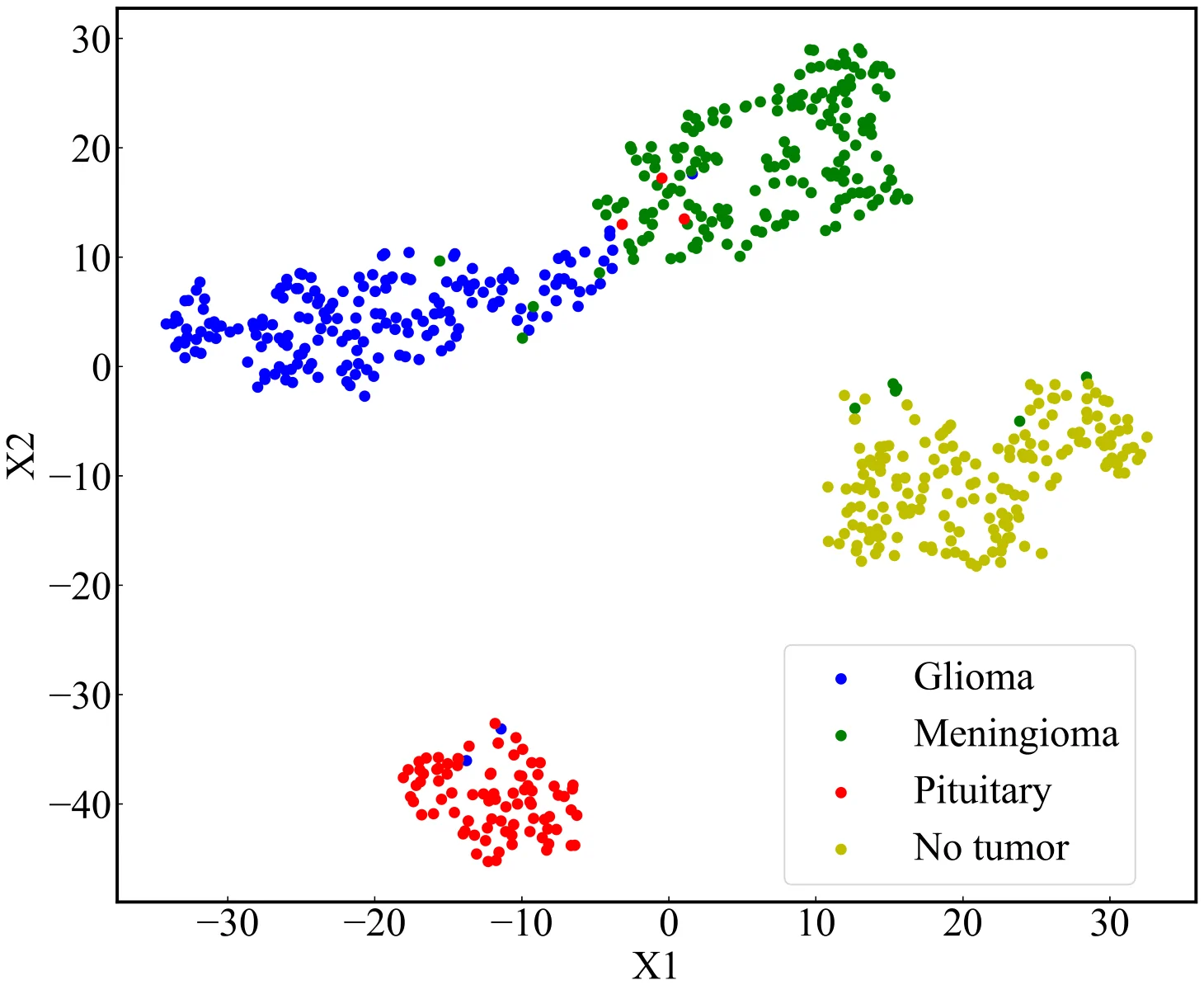
The scatter plot of the proposed model on the Kaggle dataset.

Table 13 shows the robustness of the proposed model in processing low-quality magnetic resonance images after the introduction of Gaussian noise. After introducing Gaussian noise, the model achieved an accuracy of 96.9%, which demonstrates its strong robustness and reliable performance.

**Table 13:** Performance comparison data of models on Chen dataset with Gaussian noise.

Method	Class	Precision (%)	Recall (%)	F1-score (%)	Accuracy (%)
Add Gaussian noise	Glioma	97.89	97.54	97.72	96.90
	Meningioma	91.22	95.54	93.43	
	Pituitary	100.00	96.77	98.36	

Table 14 presents the diverse metrics of the proposed model when it is evaluated on the Brain Tumor MRI dataset. This dataset, which comprises 7,023 grayscale magnetic resonance (MR) images, is a publicly available dataset on the Kaggle platform. It includes 1,621 images of gliomas, 1,645 images of meningiomas, 1,757 images of pituitaries, and 2,000 images without tumors. By examining the metrics in Table 14, it becomes evident that outstanding performance is demonstrated by the proposed model. Specifically, each performance metric of the proposed model nearly reaches 100%. Furthermore, the accuracy of the proposed model achieved 99.54%, which underscores its robust generalization capability to new and unseen datasets.

**Table 14:** Performance indicators of the proposed model on the Brain Tumor MRI dataset.

Class	Precision (%)	Recall (%)	F1-score (%)	Accuracy (%)
Glioma	100.00	98.67	99.33	99.54
Meningioma	98.71	99.67	99.19	
Pituitary	99.67	99.67	99.67	
No Tumor	99.75	100.00	99.88	

To visually see which regions the model focuses on in the brain tumor classification task, we used the Grad-CAM [47]technique to visualize it. This technique is used to help us see how brain tumors are identified and classified by the model. As shown in Fig 14, Fig 14 (a) shows pictures of various brain tumor areas before visualization and Fig 14 (b) shows heatmaps after visualization. Fig 14 (b) shows more directly the reference of this Grad-CAM technique, where the red part represents the brain tumor region.

**Fig 14:**
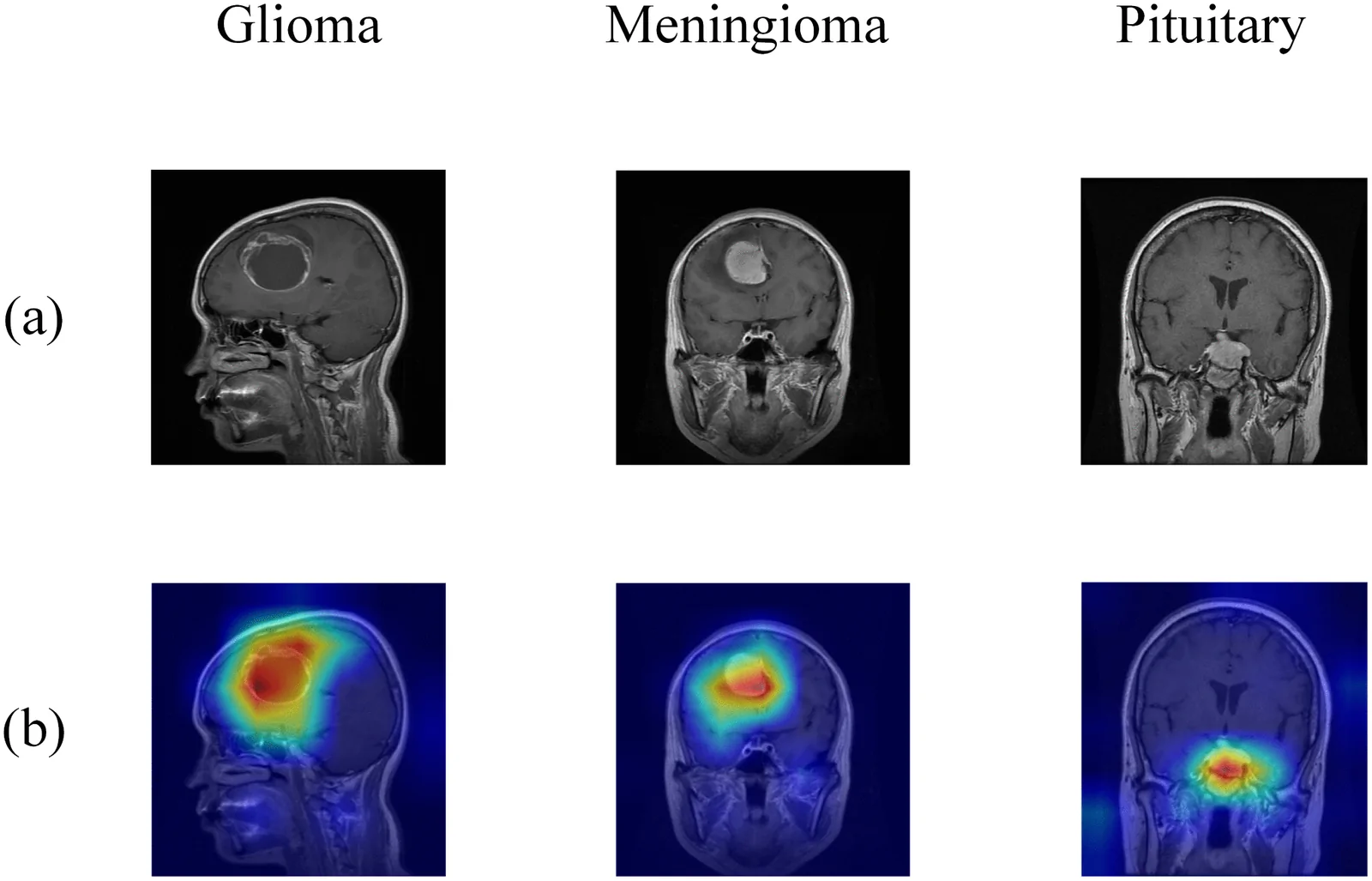
Grad-CAM visualization of different tumors. (a) brain tumor (b) visual heat map.

## 5. Conclusion

Early diagnosis of brain tumors plays a crucial role in treatment decision-making. Given the cumbersome and time-consuming nature of manual examination of brain tumors, the imperative need arises for the development of automated detection methods to aid physicians. A method for efficient detection of multiple types of brain tumors is proposed in this paper. Initially, the ConvDAtt block, featuring depthwise separable convolution, attention mechanisms, and residual connectivity, is utilized. The use of depthwise separable convolution reduces the number of parameters and improves the feature extraction capability of the proposed model. The introduced CA mechanism dynamically adjusts weight allocation based on input data characteristics, enabling the model to prioritize key features while disregarding irrelevant information. Secondly, the CI block is introduced, which is a downsampling module that combines convolution and pooling. In the CI block, the complementary nature of convolution and pooling helps to extract feature information and enables the CNN to make decisions using both local and global information while also significantly reducing the computational burden (decreasing FLOPs from 97.14G to 2.85G). Thirdly, the SPP block is introduced to capture multi-scale spatial features and enhance feature representation, which enhances model robustness. And the method’s effectiveness was verified through thorough ablation experiments. Experimental results show that the proposed method achieves 98.69% accuracy on Chen dataset and 97.25% accuracy on Kaggle dataset. These results demonstrate the competitive performance of the proposed method on both Chen and Kaggle datasets, further validating its effectiveness. With the increasing availability and diversity of medical image data, the generalization ability and robustness of deep learning models can be further improved. Therefore, future studies will focus on expanding datasets with larger-scale and more diverse samples. Moreover, external multi-center clinical validation will be explored to further evaluate the generalization capability of the proposed method. Future research directions also include multimodal image fusion, explainable artificial intelligence, and further exploration of practical deployment strategies.

## References

[1] Alyami J, Rehman A, Almutairi F, Fayyaz AM, Roy S, Saba T, et al. Tumor localization and classification from MRI of brain using deep convolution neural network and salp swarm algorithm. Cogn Comput. 2023;16(4):2036–46. doi: 10.1007/s12559-022-10096-2

[2] Ali RR, Yaacob NM, Alqaryouti MH, Sadeq AE, Doheir M, Iqtait M, et al. Learning architecture for brain tumor classification based on deep convolutional neural network: classic and ResNet50. Diagnostics (Basel). 2025;15(5):624. doi: 10.3390/diagnostics15050624 40075870 PMC11898819

[3] Almijalli M, Almusayib FA, Albugami GF, Aloqalaa Z, Altwijri O, Saad AS. Automatic active contour algorithm for detecting early brain tumors in comparison with AI detection. Processes. 2025;13(3):867. doi: 10.3390/pr13030867

[4] Ghosh A, Soni B, Baruah U. Transfer learning-based deep feature extraction framework using fine-tuned EfficientNet B7 for multiclass brain tumor classification. Arab J Sci Eng. 2023;49:12027–48. doi: 10.1007/s13369-023-08607-w

[5] Bansal T, Jindal N. An improved hybrid classification of brain tumor MRI images based on conglomeration feature extraction techniques. Neural Comput & Applic. 2022;34(11):9069–86. doi: 10.1007/s00521-022-06929-8

[6] Mukadam SB, Patil HY. Machine learning and computer vision based methods for cancer classification: A systematic review. Arch Computat Methods Eng. 2024;31(5):3015–50. doi: 10.1007/s11831-024-10065-y

[7] Beunza J-J, Puertas E, García-Ovejero E, Villalba G, Condes E, Koleva G, et al. Comparison of machine learning algorithms for clinical event prediction (risk of coronary heart disease). J Biomed Inform. 2019;97:103257. doi: 10.1016/j.jbi.2019.103257 31374261

[8] Zeinali N, Youn N, Albashayreh A, Fan W, Gilbertson White S. Machine learning approaches to predict symptoms in people with cancer: systematic review. JMIR Cancer. 2024;10:e52322. doi: 10.2196/52322 38502171 PMC10988375

[9] El-Dahshan E-SA, Hosny T, Salem A-BM. Hybrid intelligent techniques for MRI brain images classification. Digital Signal Processing. 2010;20(2):433–41. doi: 10.1016/j.dsp.2009.07.002

[10] Abd-Ellah MK, Awad AI, Khalaf AAM, Hamed HFA. A review on brain tumor diagnosis from MRI images: Practical implications, key achievements, and lessons learned. Magn Reson Imaging. 2019;61:300–18. doi: 10.1016/j.mri.2019.05.028 31173851

[11] Basheera S, Satya Sai Ram M. Classification of brain tumors using deep features extracted using CNN. J Phys: Conf Ser. 2019;1172:012016. doi: 10.1088/1742-6596/1172/1/012016

[12] Santamaria BC, Carreno EC, Barrera AG. Classification of Primary Brain Tumors Using Convolutional Neural Network (CNN). In: 2022 IEEE Colombian Conference on Applications of Computational Intelligence (ColCACI), 2022. 1–6. 10.1109/colcaci56938.2022.9905365

[13] Roy SS, Rodrigues N, Taguchi Y. Incremental dilations using CNN for brain tumor classification. Appl Sci. 2020;10(14):4915. doi: 10.3390/app10144915

[14] Sagar GV, Kumar MR, Ahammad SkH, Santhosh C. Image classification of intracranial tumor using deep residual learning technique. Multimed Tools Appl. 2023;83(19):57409–27. doi: 10.1007/s11042-023-17712-9

[15] Deepak S, Ameer PM. Brain tumor categorization from imbalanced MRI dataset using weighted loss and deep feature fusion. Neurocomputing. 2023;520:94–102. doi: 10.1016/j.neucom.2022.11.039

[16] Kang J, Ullah Z, Gwak J. MRI-based brain tumor classification using ensemble of deep features and machine learning classifiers. Sensors (Basel). 2021;21(6):2222. doi: 10.3390/s21062222 33810176 PMC8004778

[17] Al-Azzwi ZHN, Nazarov AN. Brain tumor classification based on improved stacked ensemble deep learning methods. Asian Pacific J Cancer Prevention. 2023;24:2141–8. doi: 10.31557/APJCP.2023.24.6.2141 PMC10505861 37378946

[18] Khan SUR, Zhao M, Li Y. Detection of MRI brain tumor using residual skip block based modified MobileNet model. Cluster Comput. 2025;28(4). doi: 10.1007/s10586-024-04940-3

[19] Asif S, Zhao M, Chen X, Zhu Y. BMRI-NET: A deep stacked ensemble model for multi-class brain tumor classification from MRI images. Interdiscip Sci Comput Life Sci. 2023;15:499–514. doi: 10.1007/s12539-023-00571-1 37171681

[20] Ghosh A, Soni B, Baruah U. Transfer learning-based deep feature extraction framework using fine-tuned EfficientNet B7 for multiclass brain tumor classification. Arab J Sci Eng. 2023. doi: 10.1007/s13369-023-08607-w

[21] Irfan M, Shaf A, Ali T, Farooq U, Rahman S, Nasar Faraj Mursal S, et al. Effectiveness of Deep learning models for brain tumor classification and segmentation. Computers, Materials & Continua. 2023;76(1):711–29. doi: 10.32604/cmc.2023.038176

[22] Isunuri BV, Kakarla J. Ensemble coupled convolution network for three-class brain tumor grade classification. Multimed Tools Appl. 2023;83(19):57643–59. doi: 10.1007/s11042-023-17760-1

[23] Gómez-Guzmán MA, Jiménez-Beristaín L, García-Guerrero EE, López-Bonilla OR, Tamayo-Perez UJ, Esqueda-Elizondo JJ, et al. Classifying brain tumors on magnetic resonance imaging by using convolutional neural networks. Electronics. 2023;12(4):955. doi: 10.3390/electronics12040955

[24] Trisheela S, Fernandes R, Rodrigues AP, Supreeth S, Ambika BJ, Pareek PK, et al. Brain tumour detection using VGG-Based feature extraction with modified DarkNet-53 model. Int J Biomed Imaging. 2025;2025:5535505. doi: 10.1155/ijbi/5535505 40487802 PMC12143945

[25] Tariq A, Iqbal MM, Javed Iqbal M, Iftikhar A. Transforming brain tumor detection empowering multi-class classification with vision transformers and EfficientNetV2. IEEE Access. 2025;13:63857–76. doi: 10.1109/access.2025.3555638

[26] Li J, Guan W. Patch merging refiner embedding UNet for image denoising. Inf Sci. 2023;641:119123. doi: 10.1016/j.ins.2023.119123

[27] Huang Z, Wang J, Fu X, Yu T, Guo Y, Wang R. DC-SPP-YOLO: Dense connection and spatial pyramid pooling based YOLO for object detection. Inf Sci. 2020;522:241–58. doi: 10.1016/j.ins.2020.02.067

[28] Ertosun MG, Rubin DL. Automated grading of gliomas using deep learning in digital pathology images: A modular approach with ensemble of convolutional neural networks. AMIA Annu Symp Proc. 2015;2015:1899–908. 26958289 PMC4765616

[29] Zhu H, Wang J, Wang SH, Zhang YD, Gorriz JM. ConvNet-CA: A lightweight attention-based CNN for brain disease detection. In: Vicente JMF, Alvarez-Sanchez JR, Lopez FD, Adeli H, editors. Artificial intelligence in neuroscience: Affective analysis and health applications, pt I. Cham: Springer International Publishing Ag. 2022. p. 3–12. doi: 10.1007/978-3-031-06242-1

[30] Cheng J, Huang W, Cao S, Yang R, Yang W, Yun Z, et al. Enhanced performance of brain tumor classification via tumor region augmentation and partition. PLoS One. 2015;10(10):e0140381. doi: 10.1371/journal.pone.0140381 26447861 PMC4598126

[31] Bhuvaji S, Kadam A, Prajakta B, Sameer D, Swati K. Brain Tumor Classification (MRI). Kaggle. 2020. doi: 10.34740/KAGGLE/DSV/1183165

[32] Martinez-Vega B, Tkachenko M, Matkabi M, Ortega S, Fabelo H, Balea-Fernandez F, et al. Evaluation of preprocessing methods on independent medical hyperspectral databases to improve analysis. Sensors (Basel). 2022;22(22):8917. doi: 10.3390/s22228917 36433516 PMC9693077

[33] Zhang Z, Wu H, Zhao H, Shi Y, Wang J, Bai H, et al. A novel deep learning model for medical image segmentation with convolutional neural network and transformer. Interdiscip Sci. 2023;15(4):663–77. doi: 10.1007/s12539-023-00585-9 37665496

[34] Alsaggaf W, Cömert Z, Nour M, Polat K, Brdesee H, Toğaçar M. Predicting fetal hypoxia using common spatial pattern and machine learning from cardiotocography signals. Applied Acoustics. 2020;167:107429. doi: 10.1016/j.apacoust.2020.107429

[35] Nalepa J, Marcinkiewicz M, Kawulok M. Data augmentation for brain-tumor segmentation: A review. Front Comput Neurosci. 2019;13:83. doi: 10.3389/fncom.2019.00083 31920608 PMC6917660

[36] Riehl K, Neunteufel M, Hemberg M. Hierarchical confusion matrix for classification performance evaluation. J R Stat Soc Sers C-Appl Stat. 2023;72(5):1394–412. doi: 10.1093/jrsssc/qlad057

[37] Balasuburamani K, Shanmugavel KL. Brain tumor classification in MRI using hybrid ASA-based deep learning and Masi-entropy multilayer thresholding segmentation with sunflower optimization. TS. 2025;42:223–41. doi: 10.18280/ts.420120

[38] Ishaq A, Ullah FUM, Hamandawana P, Cho D-J, Chung T-S. Improved EfficientNet architecture for multi-grade brain tumor detection. Electronics. 2025;14(4):710. doi: 10.3390/electronics14040710

[39] Sterniczuk B, Charytanowicz M. An ensemble transfer learning model for brain tumors classification using convolutional neural networks. Adv Sci Technol Res J. 2024;18:204–16. doi: 10.12913/22998624/193627

[40] Bogacsovics G, Harangi B, Hajdu A. Developing diverse ensemble architectures for automatic brain tumor classification. Multimed Tools Appl. 2024;84(30):36453–96. doi: 10.1007/s11042-024-19657-z

[41] Suliman Farhan A, Khalid M, Manzoor U. Combined oriented data augmentation method for brain MRI images. IEEE Access. 2025;13:9981–94. doi: 10.1109/access.2025.3526684

[42] Alhassan AM, Altmami NI. IV3TM: Inception V3 enabled bidirectional long short-term memory network for brain tumor classification. PLoS One. 2025;20(10):e0335397. doi: 10.1371/journal.pone.0335397 41134912 PMC12551925

[43] Guo X, Liu T, Chi Q. Brain tumor diagnosis in MRI scans images using Residual/Shuffle Network optimized by augmented Falcon Finch optimization. Sci Rep. 2024;14(1):27911. doi: 10.1038/s41598-024-77523-2 39537654 PMC11560930

[44] Dutta TK, Nayak DR, Zhang Y-D. ARM-Net: Attention-guided residual multiscale CNN for multiclass brain tumor classification using MR images. Biomedical Signal Processing and Control. 2024;87:105421. doi: 10.1016/j.bspc.2023.105421

[45] Akhmetzhanova S, Serek A, Kashayev R, Kozhamuratova A. Few-shot brain tumor classification: meta- vs metric-learning comparison. Bulletin EEI. 2025;14(5):3913–22. doi: 10.11591/eei.v14i5.10706

[46] Montoya SFÁ, Rojas AE, Vásquez LFN. Classification of brain tumors: A comparative approach of shallow and deep neural networks. SN COMPUT SCI. 2024;5(1). doi: 10.1007/s42979-023-02431-7

[47] Selvaraju RR, Cogswell M, Das A, Vedantam R, Parikh D, Batra D. Grad-CAM: Visual explanations from deep networks via gradient-based localization. Int J Comput Vis. 2020;128:336–59. doi: 10.1007/s11263-019-01228-7

